# Structural basis for RISC assembly of human Argonaute2

**DOI:** 10.1016/j.molcel.2026.04.029

**Published:** 2026-05-26

**Authors:** Huaqun Zhang, Vishal Annasaheb Adhav, Audrey C. Kehling, Andrew Savidge, Zhangfei Shen, Tian-Min Fu, Kotaro Nakanishi

**Affiliations:** 1 Department of Chemistry and Biochemistry, The Ohio State University, Columbus, OH 43210, USA; 2 Ohio State Biochemistry Program, The Ohio State University, Columbus, OH 43210, USA; 3 Center for RNA Biology, The Ohio State University, Columbus, OH 43210, USA; 4 Department of Biological Chemistry and Pharmacology, The Ohio State University, Columbus, OH 43210, USA; 5 Ohio State University Comprehensive Cancer Center, Columbus, OH 43210, USA; 6 Department of Pathology, UMass Chan Medical School, Worcester, MA 01655, USA; 7 Molecular, Cellular and Developmental Biology, The Ohio State University, Columbus, OH 43210, USA; 8 Lead contact

## Abstract

Argonaute proteins (AGOs) load small RNA duplexes and select one strand to form the RNA-induced silencing complex (RISC), but the mechanism of assembly remains unclear. We report four cryogenic-electron microscopy structures of human AGO2 bound to a small interfering RNA (siRNA) duplex, which captures previously unanticipated intermediates. Unexpectedly, only the MID-PIWI lobe secures one duplex end, while α-helix 14 probes its stability. When the N domain reaches the opposite end, the L1 hairpin and Stalk wedge into the duplex and the PAZ domain engages the guide 3′ end. This configuration peels the passenger from its 5’ end while leaving it paired to the guide seed. AGO2 completely ejects the passenger by replacing it even with partially complementary target RNAs. This target-assisted passenger ejection (TAPE) also operates for the cleaved passenger strand. Our study uncovers that mRNAs are bifunctional molecules that not only serve as targets of RISCs but also help drive RISC assembly.

## In brief

Zhang et al. report cryo-EM structures of AGO2-siRNA complexes capturing RISC assembly intermediates. They reveal MID-PIWI-mediated duplex loading, α14-guided end sensing, and stepwise passenger peeling. Target RNAs drive passenger ejection, even after cleavage, which supports target-assisted passenger ejection and a bifunctional role of mRNAs in RNAi.

## Graphical abstract

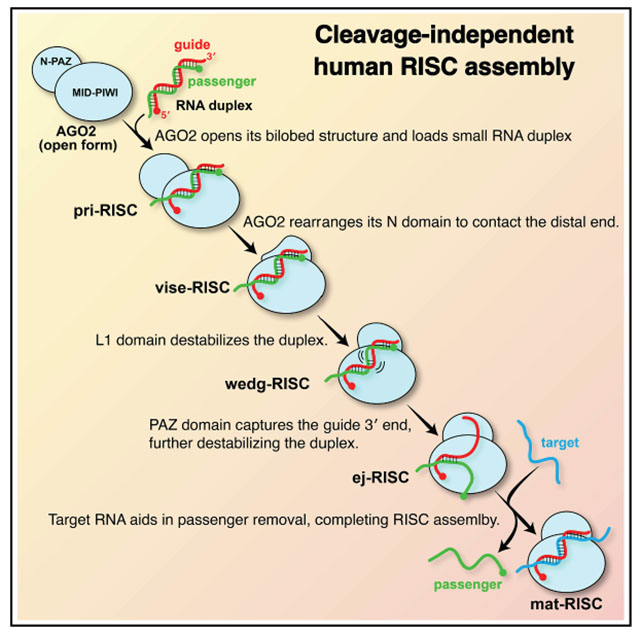

## INTRODUCTION

RNA interference (RNAi) is a post-transcriptional gene silencing mechanism that is widely observed in eukaryotes.^1^ Small interfering RNA (siRNA) and microRNA (miRNA) duplexes must be loaded into Argonaute proteins (AGOs) to form precursor RNA-induced silencing complexes (pre-RISCs), followed by passenger-strand ejection to generate mature RISCs (mat-RISCs).^2–6^ Since the strand selected from an siRNA or miRNA duplex determines which mRNAs are targeted for silencing, the assembly of RISC has been extensively studied. In flies, R2D2 recognizes the more thermodynamically stable end of long double-stranded RNA (dsRNA) substrates, while Dicer-2 cleaves the distal end, which exposes it to Ago2 and enables directional duplex loading and asymmetric guide-strand selection.^7,8^ In humans, Dicer also interacts with AGOs to deliver processed miRNA and siRNA duplexes.^9,10^ However, transfection of siRNAs, but not long duplexes, induces RNAi in Dicer-deficient murine embryonic stem cells,^11^ which indicates that Dicer is dispensable for RISC assembly and asymmetric guide-strand selection in mammals. Early studies further showed that mismatches or weaker pairings near positions 2–4 from the duplex 5’ end influence strand selection.^12,13^ This asymmetric guide selection also occurs independently of Dicer in mammals.^11,14^ Although heat shock proteins and co-chaperones enhance duplex loading in an ATP-dependent manner,^6,15^ human AGO2 can load a duplex and assemble a catalytically active RISC *in vitro* without additional factors.^16^ These findings suggest that human AGOs can distinguish thermodynamic stability differences between duplex ends, but the molecular mechanism remains elusive due to the lack of a pre-RISC structure.

A widely accepted rule for guide-strand selection states that the MID domain preferentially recognizes adenosine (A) or uridine (U) at guide nucleotide position 1 (g1) over cytidine (C) or guanosine (G) through the nucleotide-specificity loop.^17^ Another rule is that the thermodynamically less stable end of a duplex tends to fray, which allows the strand whose 5’ end is captured by the MID domain to become the guide strand.^8,18^ However, the relationship between these two rules remains unclear. Although human AGOs eject the intact passenger strand,^19^ how AGOs unwind ~22-nt siRNA or miRNA duplexes spanning roughly two helical turns and eject the passenger strand remains unknown. Thus, despite its importance for RNAi-based therapeutics, the structural basis of RISC assembly has not been elucidated.

Since the discovery of RNAi, miRNAs and siRNAs have been thought to primarily target mRNAs,^20–28^ except in cases of target-directed miRNA degradation (TDMD), where specific mRNAs trigger miRNA degradation.^29–35^ Because mRNAs interact with mat-RISCs in the cytoplasm, where pre-RISCs are also present,^36,37^ they could potentially influence RISC assembly. However, little is known about this possibility, except that target RNA abundance correlates with the stability of cognate miRNAs.^38,39^

Here, cryogenic-electron microscopy (cryo-EM) structures provide stepwise views of duplex loading, passenger unwinding, and passenger ejection, which reveal that RISC assembly involves extensive conformational changes. Our structural and biochemical analyses further show that target RNAs promote the formation of mat-RISCs by facilitating passenger ejection, which highlights an unexpected role of mRNAs in RNAi.

## RESULTS

### Overall structure of primary RISC

**Figure 1.**
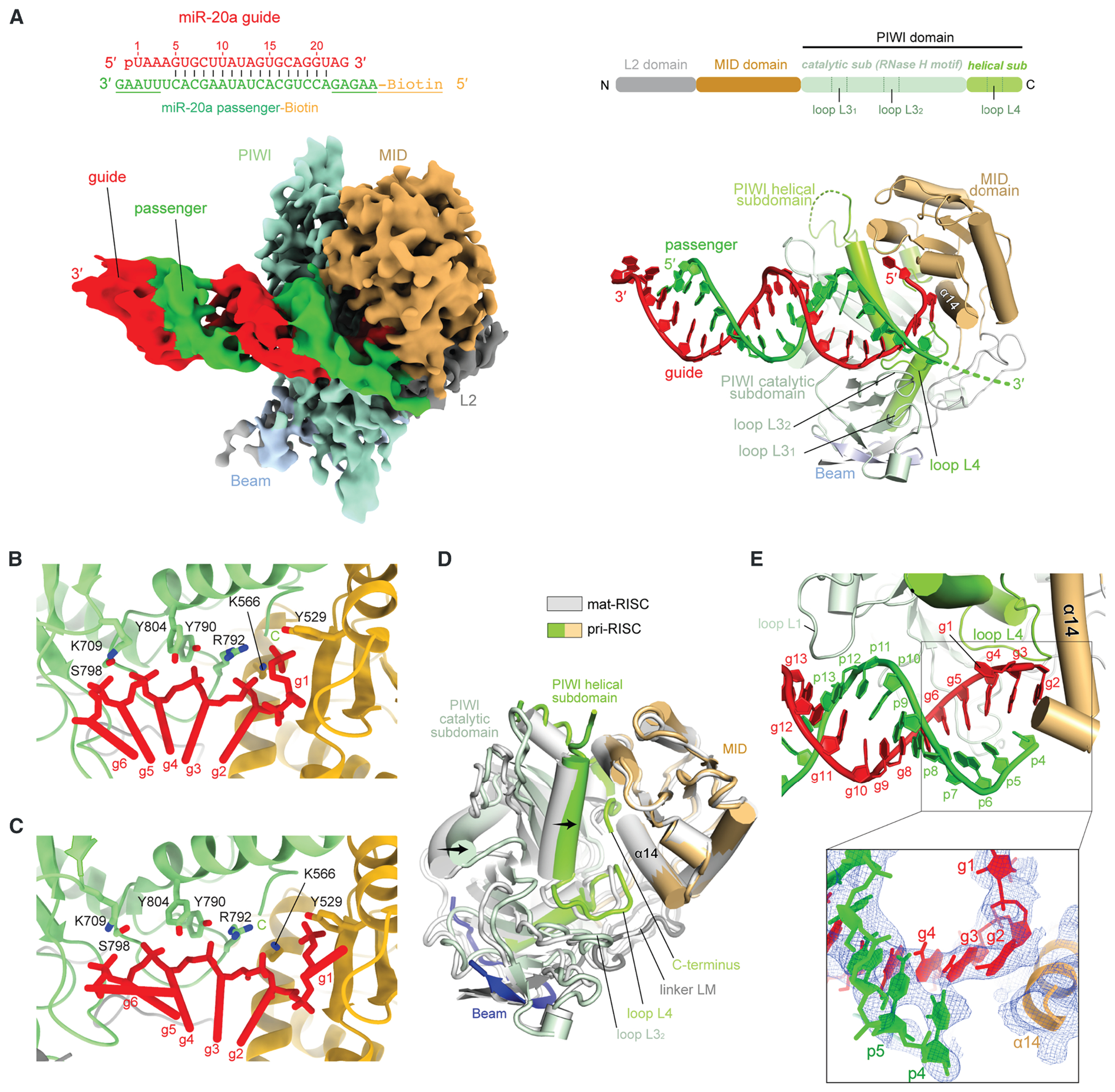
One-end recognition of the small RNA duplex by AGO2 in pri-RISC (A) Overall structure of pri-RISC. (Top left) Guide and passenger strands used for structure determination; disordered nucleotides are underscored. (Top right) Domain architecture of L2, MID, and PIWI domains. (Bottom) Cryo-EM map and model of pri-RISC. (B and C) Guide-strand recognition by pri-RISC (B) and by mat-RISC (PDB: 4OLA) (C). (D) Arrangement of the MID and PIWI domains in pri-RISC and mat-RISC with MID domains superposed. (E) Base pairing between the passenger 3′ end and the guide 5′ end in pri-RISC. (Inset) Cryo-EM density for the guide-passenger duplex. See also Figures S1–S4; Table S1; Video S1.

To capture different pre-RISC states, we incubated semi-purified human AGO2 with a miR-20a duplex whose passenger strand was biotinylated at the 5’ end, at either 4°C or room temperature (21°C) (Figures 1A and S1A; Table S1). Divalent cations were depleted to prevent passenger cleavage.^40,41^ A denaturing gel confirmed that both guide and passenger strands remained intact (Figures S1B and S1C). Consistently, the duplex-bound AGO2 did not cleave the passenger strand in either the presence or absence of 5 mM Mg^2+^ for, respectively, 3 days at 4°C or 1 h at 21°C (Figures S1D and S1E), which indicated that most complexes remained in the pre-RISC state. Cryo-EM analysis yielded well-defined 2D classes at both temperatures, and we determined a 3.79 Å structure from the 4°C dataset (Table 1; Figures S2 and S3A; Video S1).

Unexpectedly, the N-PAZ lobe was completely disordered and AGO2 engaged only the MID and PIWI domains to capture one end of the duplex through shape recognition (Figure 1A). The beam, which runs across the bottom of RISC,^5^ appears to function as a hinge between the N-PAZ and MID-PIWI lobes. The distal end of the duplex remained solvent-exposed. This accessibility was validated by footprinting with interferon-stimulated gene 20 kDa (ISG20), a 3’→5’ exonuclease that we found can degrade dsRNAs (Figures S1F and S1G), although it was previously reported to act on single-stranded (ss) RNAs.^42,43^ When loaded onto AGO2, a 23-nt ss miR-20a was trimmed to 18–23 nt because its 3’ end is captured by the PAZ domain (Figure S1H),^43^ whereas the same guide within an siRNA-like duplex was trimmed to 10–17 nt (Figure S1H), which is consistent with the guide 3’ end being largely solvent-exposed. We named this state of duplex-bound AGO “primary RISC (pri-RISC, or state i)” (Figure S1A).

The pri-RISC structure resembles the open conformation adopted by mature AGO2-RISC when only the MID and PIWI domains engage an extensively propagated guide-target duplex (Figure S4A).^44^ However, whereas the target-bound complex positions the guide-target duplex near the catalytic tetrad, pri-RISC keeps the guide-passenger duplex distant from the active site, which indicates that the two structures represent distinct conformations despite their similar overall architecture.

### AGO2 uses α14 to select the guide strand

In pri-RISC, AGO2 flips g1 to stack with Y529 while it recognizes the phosphate backbone of g2–g6, which indicates that the guide seed is already engaged during duplex loading, as in mat-RISC (Figures 1B and 1C).^45^ Compared with mat-RISC, the PIWI domain in pri-RISC is positioned ~4 Å closer to the MID domain, which places α14 between the guide and passenger strands (Figures 1A and 1D). This rearrangement disrupts base pairing from g1 to g4 (Figure 1E). Consistent with this, densities for passenger nucleotide positions 1–3 (p1–p3) and the 2-nt 3’ overhanging end are unresolved, and p4 density is weak. Superposition of an ideal A-form duplex onto the AGO2-bound duplex revealed steric clashes with α14, loops L3_2_ and L4, and linker LM,^46^ which suggests that these elements cooperatively pry open the duplex end (Figures S4B and S4C). These observations indicate that pri-RISC represents a state in which AGO2 probes the thermodynamic stability of the captured duplex end.

**Figure 2.**
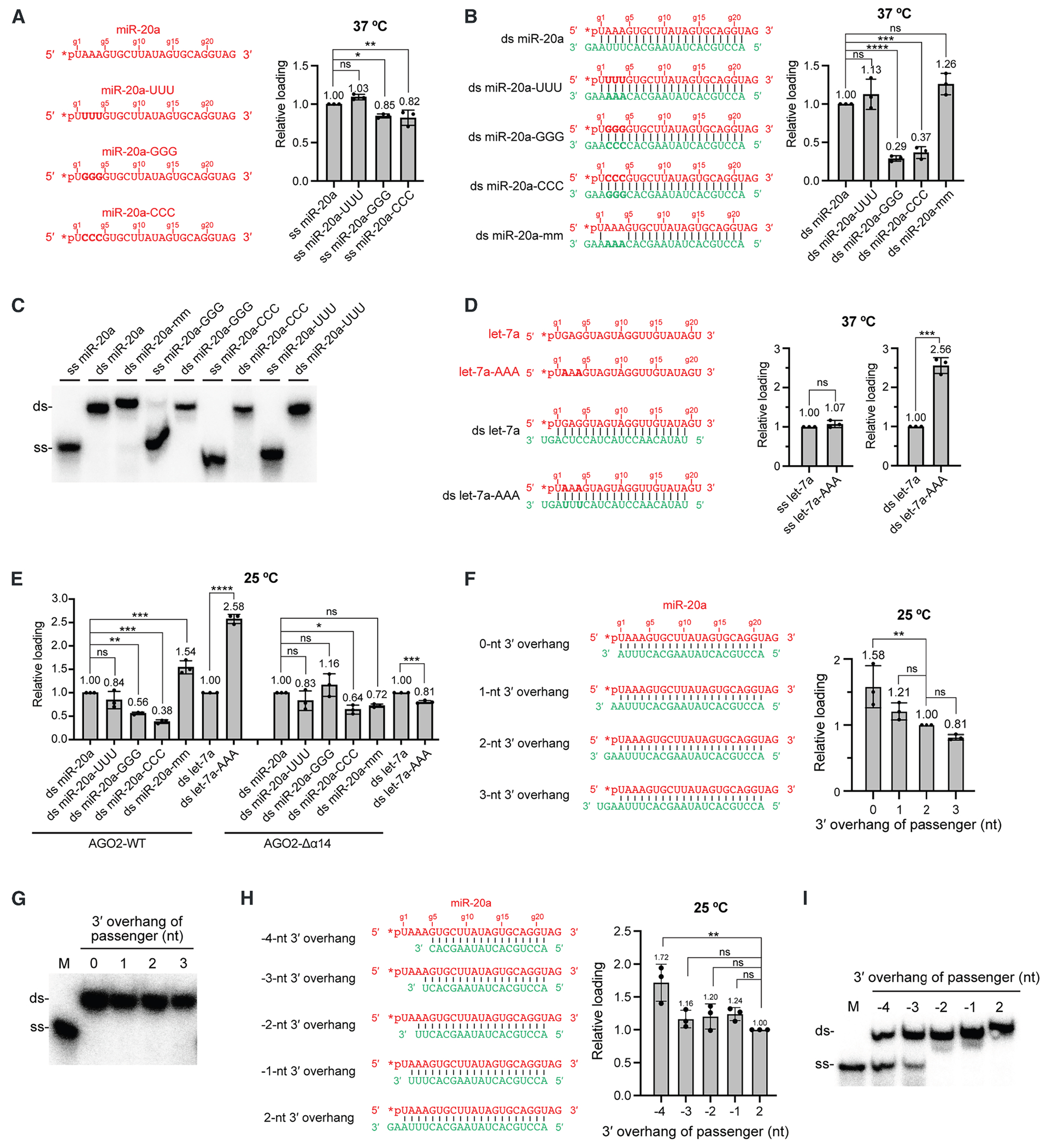
α14-dependent asymmetric guide-strand selection (A and B) *In vitro* loading of 5′-end-radiolabeled ss (A) or ds (B) miR-20a variants into purified recombinant FLAG-AGO2 at 37°C. (C) Half of the RNAs recovered in (A and B) were reannealed and resolved on a 16% native gel at 4°C. (D) *In vitro* loading of ss and ds let-7a variants into recombinant FLAG-AGO2 at 37°C. (E) Loading of the indicated RNAs into immunopurified FLAG-AGO2 WT or the α14 mutant at 25°C. (F and H) Loading of miR-20a duplexes with 0–3-nt (F) or −1 to −4-nt (H) passenger 3′ overhanging ends into recombinant FLAG-AGO2 at 25°C. (G and I) Half of the RNAs recovered in (F and H) were reannealed and resolved on a 16% native gel at 4°C. M, ss miR-20a marker. Data represent mean ± SD (*n* = 3). * *p* < 0.05, ** *p* < 0.01, *** *p* < 0.001, and **** *p* < 0.0001; ns, not significant (analysis by one-way ANOVA for A, B, D, E, F, and H; unpaired *t* test for D). See also Figures S4 and S5; Table S1.

To test this hypothesis, we performed *in vitro* loading assays using purified FLAG-AGO2 in the absence of Mg^2+^ to prevent passenger cleavage. FLAG-AGO2 was incubated at 37°C with one of four ss guides: 23-nt miR-20a or variants containing UUU, GGG, or CCC substitutions at g2–g4. Anti-FLAG pulldown assays showed similar binding for all ss guides (Figures 2A and S5A; Table S1). In contrast, when paired with fully complementary passengers, ds miR-20a-UUU bound AGO2 comparably to ds miR-20a, whereas ds miR-20a-CCC and ds miR-20a-GGG bound less efficiently (Figure 2B; Table S1). The miR-20a duplex also bound slightly better when annealed to a p2–p4 mismatched passenger than to a fully complementary strand (Figure 2B; Table S1). Importantly, all duplexes remained double-stranded after binding, which indicated that duplex loading alone does not autonomously generate mat-RISC *in vitro* (Figure 2C). Similarly, increasing the AU content at the end of a let-7a siRNA-like duplex enhanced AGO2 binding, whereas no difference was observed for the corresponding ss guides (Figures 2D and S5B; Table S1). These results indicate that AGO2 preferentially loads siRNA duplexes by recognizing the thermodynamically less stable end.

Because α14 partially separates the guide and passenger strands (Figure 1A), we hypothesized that it functions as the duplex-end stability sensor. Immunopurified FLAG-AGO2-WT (wild type) from HEK293T cells showed the same duplex-binding preference as recombinant AGO2 when incubated with miR-20a or let-7a duplexes at 25°C (Figures 2E and S5C–S5E). In contrast, a mutant lacking part of α14 (T556–N562; FLAG-AGO2-Δα14) bound all duplexes similarly regardless of GC content at the captured end (Figures 2E and S5C–S5E). The same trend was observed at 37°C, although the asymmetry was modestly reduced (Figures S5F–S5H). These results demonstrate that α14 functions as the thermodynamic stability sensor underlying AGO2 guide-strand selection.

### Impact of the passenger 3’ overhanging end on RISC assembly

During RISC assembly, the strand bearing a 3’ overhanging end is preferentially selected as the guide,^47–49^ but the contribution of the passenger 3’ overhanging end remains unclear. In the pri-RISC structure, density corresponding to the passenger 3’ overhanging end is disordered (Figure 1E). Consistent with this observation, binding assays showed that AGO2 associated with a larger fraction of duplexes when the passenger carried a shorter 3’ overhanging end (Figures 2F and S5I; Table S1). All AGO2-associated duplexes remained double-stranded (Figure 2G), which indicated that the passenger 3’ overhanging end primarily affects duplex loading rather than subsequent passenger dissociation.

To further examine this effect, we tested siRNA duplexes whose passenger strands carried −4-, −3-, −2-, or −1-nt 3’ overhanging ends (Figure 2H; Table S1). Duplexes with −1- to −3-nt overhanging ends loaded with efficiencies comparable to that of the canonical 2-nt overhanging end, whereas the −4-nt overhanging end enhanced loading (Figures 2H and S5J). This trend parallels the improved loading observed for duplexes containing mismatches at p2–p4 (Figure 2B). Notably, duplexes bearing −4- or −3-nt 3’ overhanging ends lost passenger-guide pairing after AGO2 association (Figure 2I), whereas the p2–p4 mismatched duplex remained double-stranded (Figure 2C). These results suggest that the presence of p1–p4 and a 2-nt 3’ overhanging end helps maintain the guide-passenger duplex in states following pri-RISC, even when these nucleotides do not pair with the guide strand.

### L1 hairpin and Stalk initiate duplex distortion

**Figure 3.**
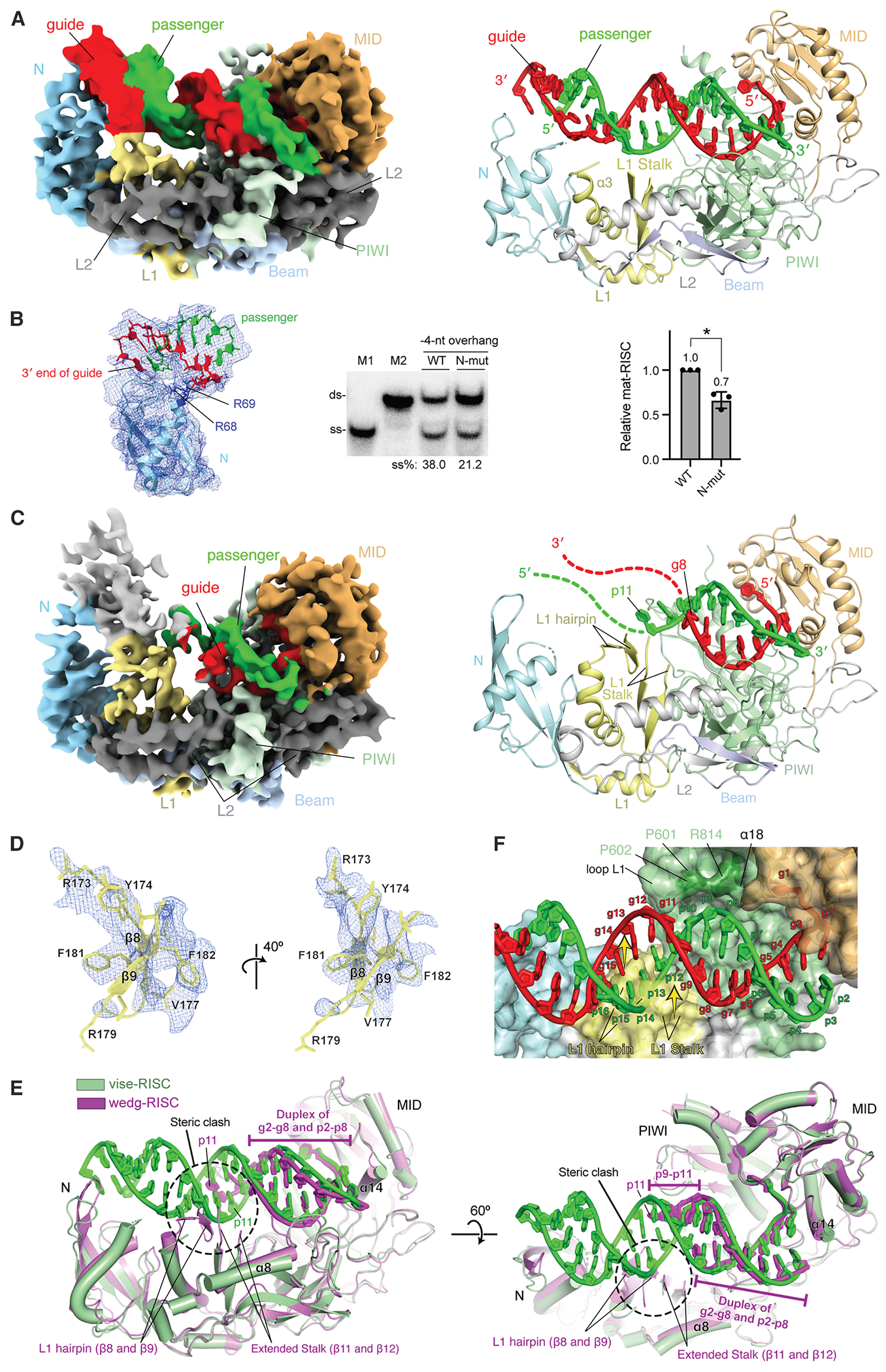
Cryo-EM structures of vise- and wedg-RISCs (A) Cryo-EM maps and models of vise-RISC. Colors are as in Figure 1A; the N and L1 domains missing in pri-RISC are colored cyan and yellow, respectively. (B) Positively charged residues in an N-domain loop facilitate RISC maturation. (Left) Cryo-EM density for the N domain and distal end of the duplex in vise-RISC (K62 and K65 lie on the disordered loop). (Middle) 16% native gel of RNAs reannealed after *in vitro* loading at 37°C using immunopurified FLAG-AGO2 WT or the N mutant; the guide RNA was 5′-32P-labeled. M1 and M2 denote ss and ds miR-20a markers, respectively. (Right) Quantification of mat-RISC (ss band) relative to pre-RISC (ds band). Data are mean ± SD (*n* = 3); * *p* < 0.05 by unpaired *t* test. (C) Cryo-EM map and model of wedg-RISC. Colors are as in (A). (D) L1 hairpin of wedg-RISC with cryo-EM density from (C). (E) Superposition of vise- and wedg-RISCs aligned on the MID domain. Steric clashes between the duplex of vise-RISC (green) and the L1 hairpin and Stalk of wedg-RISC (magenta) are indicated. (F) Steric clash between the duplex of vise-RISC (green and red) and AGO2 of wedg-RISC (surface model) when aligned on the MID domain. Yellow arrows indicate movement of the L1 hairpin and Stalk during the transition from vise- to wedg-RISC. See also Figures S2 and S3; Table S1; Videos S2 and S3.

From the 4°C dataset, we reconstructed two additional structures with 2D classes distinct from pri-RISC (Table 1; Figures S1A and S2). In one structure, the N domain together with portions of the L1 and L2 domains form a vise-like arrangement around the duplex, which is in addition to the MID and PIWI domains. In this conformation, the PAZ domain, the L1 hairpin (β8–β9), the L1 Stalk (β10–β12), and helix α7 of the L2 domain are disordered (Figure 3A; Video S2). The N domain is positioned near the distal end of the duplex. Although its local resolution is lower than the rest of AGO2 (Figure S3B), the density suggests that a lysine- and arginine-rich loop approaches the phosphate backbone of the guide strand (Figure 3B). Consistent with this arrangement, mutation of these residues reduced RISC maturation when AGO2 was incubated with a −4-nt overhanging end duplex that otherwise supports autonomous passenger ejection by AGO2-WT (Figures 2I and 3B; Table S1). Given the established role of the N domain in passenger ejection,^50^ this conformation likely represents a duplex-stabilizing intermediate preceding strand separation. We therefore designate this state “vise-RISC” (state ii).

The second structure shows a similar overall architecture but with the L1 hairpin and a larger portion of the L1 Stalk ordered and positioned adjacent to the duplex (Figures 3C, 3D, S2, and S3C). Because these elements occupy a region incompatible with the intact duplex observed in vise-RISC, we designate this state “wedging RISC” (wedg-RISC or state iii) (Figure S1A). Structural superposition indicates that the L1 hairpin and Stalk overlap with the central duplex region observed in vise-RISC, particularly around p12–p16 (Figures 3E and 3F), which suggests that these elements stabilize a locally disrupted duplex during the transition from vise-RISC to wedg-RISC. This interpretation agrees with reports that mismatches within p12–p15 enhance duplex unwinding.^51^ In contrast, loop L1 and helix α18 of the PIWI domain remain positioned near base pairs p8–p10 and g8–g10, maintaining these interactions (Figure 3F). Consequently, the guide-passenger duplex bends beyond g8 and p11, while the distal region remains continuous but poorly resolved (Figure 3C; Video S3).

### Passenger-strand ejecting state

**Figure 4.**
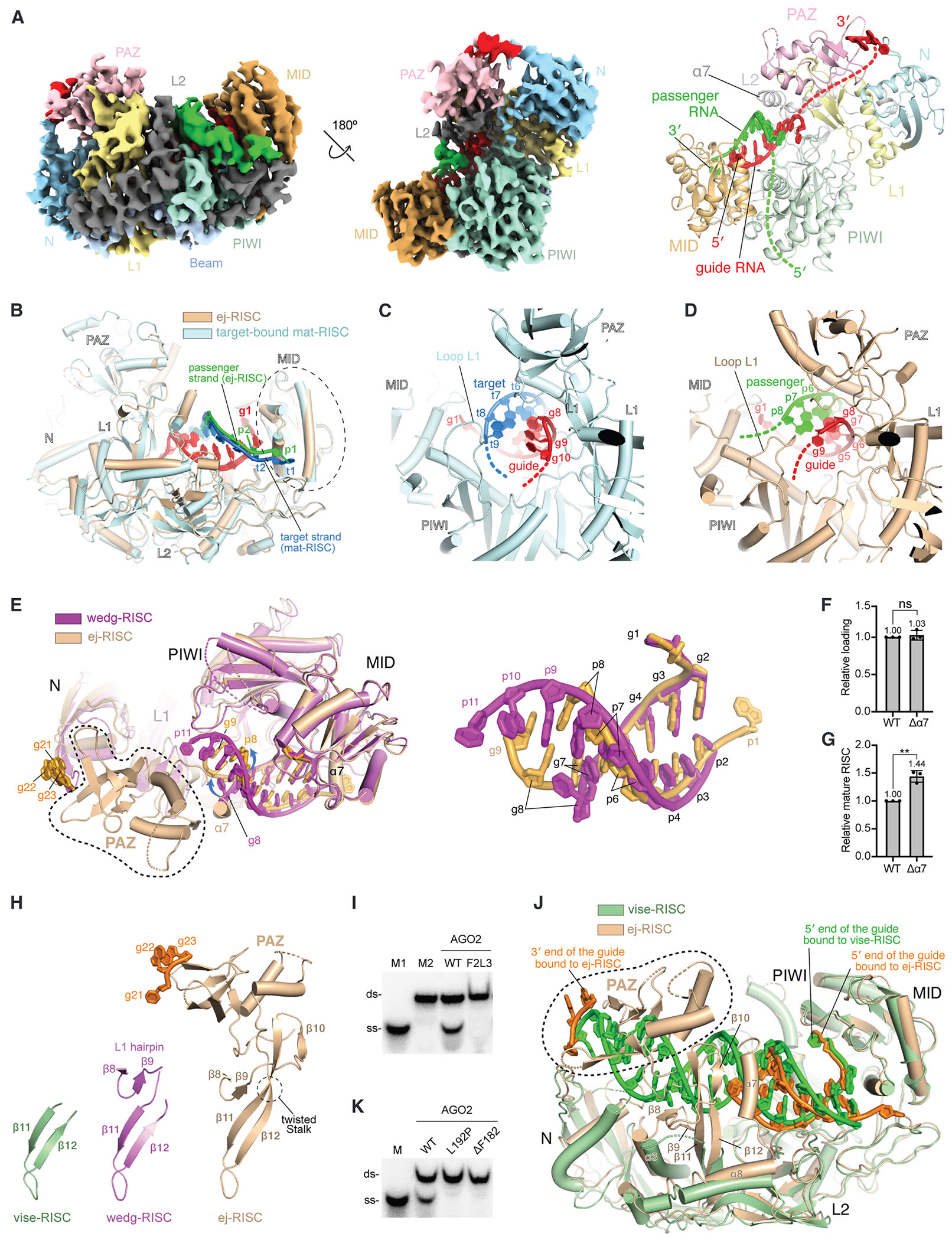
ej-RISC structure and conformational change from wedg-RISC (A) Cryo-EM map and model of ej-RISC. (B) Superposition of ej-RISC and target-bound mat-RISC on their MID domain. Both guide strands bound to ej-RISC and target-bound mat-RISC are colored in red. The passenger strand of ej-RISC and the target strand bound to mat-RISC are colored in green and blue, respectively. (C and D) Different arrangements of loop L1 between target-bound mat-RISC (C) and ej-RISC (D). (E) (Left) Superposition of wedg- and ej-RISCs aligned on the MID domain; the PAZ domain of ej-RISC is highlighted (dotted circle), and duplex movements are indicated by blue arrows. (Right) Duplexes bound to wedg- and ej-RISCs are colored magenta and orange, respectively. (F and G) In vitro loading of miR-20a duplexes into immunopurified FLAG-AGO2 WT or Δα7 (C) and quantification of RISC maturation (D) at 37°C. Data are mean ± SD (*n* = 3); ** *p* < 0.01, ns, not significant (analysis by unpaired *t* test). (H) Comparison of the L1 hairpin (β8–β9) and Stalk (β10–β12) in vise-, wedg-, and ej-RISCs. (I) Duplex loading assay using F2L3 (16% native gel); M1 and M2 are ss and ds miR-20a markers, respectively; guide RNAs were 5′-32P-labeled. (J) Steric clash between the duplex of vise-RISC (green) and AGO2 of ej-RISC (wheat) when aligned on the MID domain. (K) Duplex loading assay using AGO2 syndrome mutants, L192P and ΔF182 (16% native gel). All assays were triplicated. See also Figures S6–S8; Table S1; Video S4.

An additional 2D class was observed exclusively when AGO2 was incubated with the duplex at 21°C, which allowed the determination of a cryo-EM structure at 3.28 Å resolution (Table 1; Figures S1A, S6, and S7A). Unlike pri-, vise-, and wedg-RISC, this structure resolves all AGO2 domains and displays the bilobed architecture. Density corresponding to the passenger strand is retained only for its 3’ side, p1–p8, which remain base paired with the guide seed region (g2–g8), whereas the remainder of the passenger strand lacks interpretable density (Figure 4A; Video S4). This asymmetric density pattern indicates that passenger-guide base pairing is disrupted preferentially from the passenger 5’ region. The same siRNA duplex remained stably associated with AGO2 at 25°C and 37°C (Figures 2C, 2G, and 2I), which argues against the possibility that, at 21°C, AGO2 first formed mat-RISC and subsequently rebound the passenger strand as a target RNA. To compare this state with target-bound mat-RISC, we purified a homogeneous AGO2-RISC complex loaded with 23-nt miR-20a, which was incubated with an 18-nt target RNA containing mismatches at t10–t11 to avoid cleavage, and then determined its cryo-EM structure at 3.0 Å resolution (Figures S8A–S8C; Table 1; Video S5). The density clearly resolves base pairing between g2–g9 and t2–t9, whereas the remaining density extends toward the PAZ domain (Figure S8D). The low-pass Gaussian-filtered map shows a density of the 3’ supplementary region of the guide-strand pairing with the target RNA (Figure S8E). In contrast, the bilobed structure described above retains density only for the guide 3’ supplementary region (Figure S8F). Structural superimposition further reveals distinct arrangements of both the duplex and AGO2 domains (Figure 4B), which indicates that AGO2 recognizes the passenger and target strands differently. We therefore refer to the bilobed conformation observed at 21°C as the “passenger-ejecting RISC (ej-RISC or state iv)” (Figure S1A).

The target-bound mat-RISC arranges loop L1 outward, which allows both the guide and the target strands to pass through the nucleic acid-binding channel (Figure 4C). In contrast, the ej-RISC locates loop L1 between the guide and passenger strands, which splits the duplex and allows only the guide strand to pass through the channel (Figure 4D). This conformation of ej-RISC sequesters the 3’ supplementary region from pairing with the passenger strand.

### Conformational changes from vise- to ej-RISC through wedg-RISC

Comparison of wedg- and ej-RISC revealed several conformational differences. In ej-RISC, helix α7 becomes ordered and is adjacent to the minor groove of the seed-region duplex (Figure 4A), which positions the duplex closer to the PIWI domain than in wedg-RISC (Figure 4E). Substitution of α7 with pentaglycine^52^ did not affect duplex loading (Figures 4F and S5C; Table S1), which is consistent with pri-, vise-, and wedg-RISC structures where α7 is disordered (Figures 1A, 3A, and 3C). However, the α7 mutant exhibited increased passenger-ejection efficiency (Figure 4G; Table S1), which indicated that α7 helps maintain base pairing between the passenger strand and the guide seed region in ej-RISC.

The Stalk region also changes markedly. Whereas it adopts an extended conformation in vise- and wedg-RISCs, it becomes twisted in ej-RISC, which reorients the PAZ domain toward the guide 3’ end (Figure 4H). This rearrangement suggests that PAZ capture of the guide 3’ end promotes the wedg- to ej-RISC transition during slicer-independent assembly. Consistently, an AGO2-F2L3 mutant defective in 3’-end binding at the PAZ domain^53^ loaded the −4-nt overhanging end duplex efficiently but failed to eject the passenger strand (Figure 4I; Table S1), which indicated that PAZ-mediated 3’-end capture is indispensable for intact passenger ejection.

Because density for the post-seed duplex region is poorly resolved in wedg-RISC (Figure 3C), we compared vise- and ej-RISC to examine how duplex destabilization occurs. In ej-RISC, the PAZ domain, L1 hairpin, upper Stalk (β10–β12), and helix α7 are all resolved, whereas these elements are disordered in vise-RISC. Structural superposition shows that these elements overlap with regions corresponding to p14–p18 and g8–g19 of the duplex in vise-RISC (Figure 4J), which indicates that their ordering disrupts base pairing along one side of the duplex during the vise- to ej-RISC transition without passenger cleavage (the slicer-dependent pathway will be discussed later).

Mutations in the L1 hairpin and Stalk are associated with Argonaute syndrome, a neurodevelopmental disorder.^54–56^ Our recent work showed that AGO1 syndrome mutations ΔF180 and L190P, which are located in β9 of the L1 hairpin and β10 of the Stalk, respectively, severely impair passenger ejection.^57^ Corresponding AGO2 mutants (ΔF182 and L192P) loaded the −4-nt overhanging end duplex comparably to WT but failed to eject the passenger strand (Figure 4K; Table S1), which supports a key role for the L1 hairpin and Stalk in duplex destabilization during RISC assembly.

### Conformational changes from ej- to mat-RISC

From the same grid used to obtain ej-RISC (prepared at 21°C), we identified particles corresponding to another bilobed conformation and reconstructed a structure at 3.45 Å resolution that contains density for the guide strand but not the passenger strand (Figures S6, S7B, and S7C; Table 1; Video S6). Because duplex-loaded AGO2 was purified via a biotin tag on the passenger 5’ end (Figure 1A), these passenger-lacking particles are unlikely to result from purification artifacts. Cryo-EM studies have shown that bound partners can dissociate during vitrification due to the thin liquid film, blotting, or the air-water interface.^58,59^ We therefore interpret this structure as mat-RISC generated by passenger loss from ej-RISC during vitrification. Consistently, mat-RISC-like 2D classes were not detected in datasets collected from grids prepared at 4°C, where ej-RISC particles were absent.

**Figure 5.**
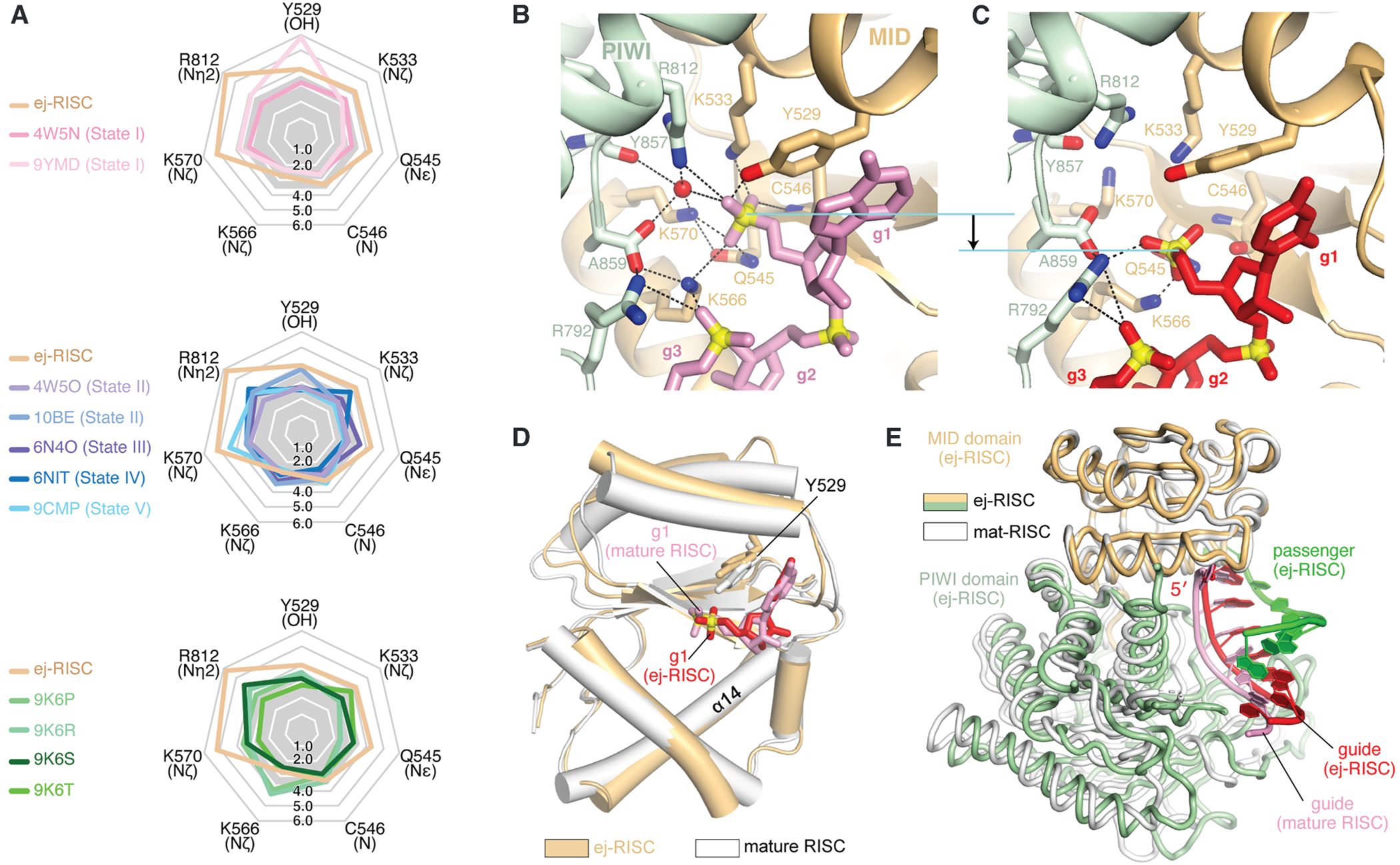
Passenger ejection is required to complete RISC assembly (A) Distances (Å) between the guide 5′ monophosphate and surrounding residues. ej-RISC is compared with state I (top), states II–V (middle), and the open conformation of mat-RISC (bottom). PDB IDs are color coded; distances within 3.5 Å are shaded gray. (B and C) Recognition of the guide 5′ end in mat-RISC (PDB: 4OLA) (B) and ej-RISC (C). MID and PIWI domains are colored wheat and green, respectively; guide RNAs are pink and red. Water is shown as a red sphere and hydrogen bonds as dotted lines. (D) Superposition of the MID domains of ej- and mat-RISCs (PDB: 4OLA). Y529 stacking with g1 is shown as sticks; only g1 nucleotides are displayed for clarity. (E) Conformational change upon passenger ejection. ej-RISC (colors as in B and C) is superposed on mat-RISC (white) via the MID domain; the ej-RISC passenger strand is shown in green. See also Figures S6–S9; Table S1; Videos S5–S7.

In mat-RISC, helix α7—positioned adjacent to the seed duplex in ej-RISC—shifts to a location between g6 and g7, which exposes nucleobases g2–g6 to solvents, as previously reported.^45,60^ The transition from ej- to mat-RISC also involves a global rearrangement in which the N-PAZ lobe and PIWI domain move closer to the MID domain, which enables a deeper placement of the guide 5’ monophosphate into the MID-PIWI interface (Video S7). This configuration forms an extensive hydrogen-bonding network around the 5’ monophosphate (Figure 5A), which is a hallmark of previously reported AGO2 mat-RISC structures that include the target-free state (state I), complexes paired at t2–t8 (state II), t2–t8 and t13–t16 (state III), t2–t8 and t13–t21 (state IV), t2–t21 (state V) (Figures S9A–S9E; Table S1),^45,61–65^ and even the open conformation (Figure S4A).^66^ Across mat-RISC states I–V, the central channel progressively widens as the guide-target duplex extends, whereas the relative positions of the MID and PIWI domains remain largely unchanged (Figures S9F and S9G). In all these states, the guide 5’ monophosphate maintains extensive hydrogen bonding with the MID and PIWI domains, regardless of target pairing (Figure 5B). In contrast, in ej-RISC, the 5’ mono-phosphate occupies a shallower position and forms fewer hydrogen bonds (Figures 5C and 5D), which reflects the constraints imposed by the passenger strand that limit access of the guide 5’ end to the MID-PIWI pocket (Figure 5E). These observations indicate that complete passenger dissociation is required for full accommodation of the guide 5’ end within its binding pocket.

### TAPE

**Figure 6.**
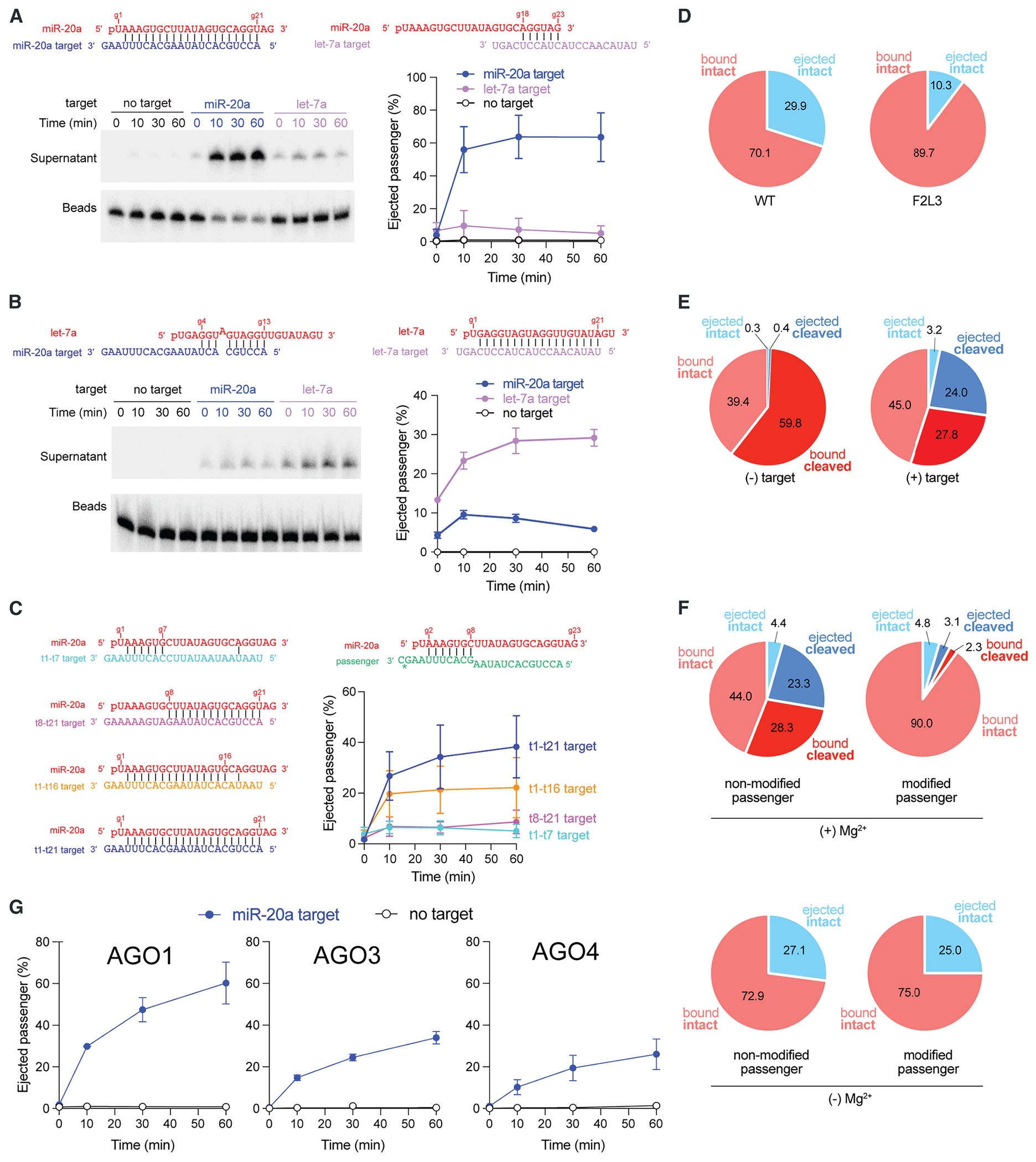
Target-assisted passenger ejection drives the ej- to mat-RISC transition (A and B) Passenger-ejection assays with AGO2 in the absence of Mg^2+^. (Top) Base pairing between the guide (red) and targets (blue or pink). (Bottom) Representative gel and time course of passenger ejection after AGO2 was preloaded with miR-20a (A) or let-7a (B) duplexes. (C) Determination of target requirements for TAPE. (Top right) Guide-passenger pairing observed in the ej-RISC structure (Figure 4A); the asterisk indicates ^32^P label at the passenger 3′ end. (Left) Pairing between the guide (red) and four target RNAs. (Bottom right) Time course of passenger ejection by each target. (D) Passenger ejection by AGO2 WT (left) and the F2L3 mutant (right) without Mg^2+^ at 37°C. (E) Passenger ejection by AGO2 in the presence or absence of target with Mg^2+^ at 37°C. (F) Passenger ejection by AGO2 loaded with unmodified or modified duplexes in the presence (top) or absence (bottom) of Mg^2+^ at 37°C. (G) Passenger ejection assays using AGO1, AGO3, and AGO4 without Mg^2+^ at 37°C. Assays were triplicated. Data in (A–C and G) are mean ± SD; (D–F) show means. See also Figure S10; Table S1.

The structure of ej-RISC suggests that the passenger strand stably pairs with the guide seed region (Figure 4A). Indeed, incubation of purified AGO2 with an siRNA duplex of miR-20a at 37°C did not induce passenger ejection (Figure 6A), which indicates that pre-RISC can progress to ej-RISC but cannot complete assembly on its own. However, addition of a target RNA complementary to miR-20a efficiently triggered passenger release (Figure 6A). A let-7a target, whose 6 nt were complementary to miR-20a, provided limited stimulation (Figure 6A). We repeated the experiment using the duplex, whose guide was radiolabeled at its 5’ end, which showed no guide ejection upon addition of the complementary target (Figure S10A), thus indicating that target RNAs eject the passenger strand but not the guide strand. Incubation of the siRNA duplex with the target RNA in the absence of AGO2 did not replace the passenger strand with the target, which indicated that the observed target-assisted passenger ejection (TAPE) required AGO2 (Figure S10B). When AGO2 was preloaded with a let-7a duplex, the let-7a target efficiently promoted passenger ejection (Figure 6B). The miR-20a target, which formed a 9-nt pairing with let-7a, resulted in weaker passenger ejection (Figure 6B). Thus, the efficiency of passenger release appeared to depend on the degree of the complementarity between the guide and target RNAs.

To understand TAPE’s mechanism, we tested four different targets. A t2–t7 target, complementary only to g2–g7, slightly promoted passenger release (Figures 6C and S10C), which was consistent with our structural observation that the passenger strand blocks g2–g7 in ej-RISC (Figure 4A). A t8–t21 target, complementary to g8-g21, was also ineffective (Figure 6C), as it could not propagate pairing into the seed region once annealed to g8–g21. In contrast, a t1–t16 target showed moderate passenger ejection, which was further enhanced when complementarity was extended to t17–t21 (Figure 6C; Table S1). These results indicate that TAPE initiates target pairing in the accessible post-seed region of the ej-RISC guide and propagates the guide-target duplex toward the seed region, thereby displacing the passenger strand. If this model is true, being in the ej-RISC state must be required for efficient TAPE operation. Repeating the passenger-ejection assay using the F2L3 mutant resulted in a significant reduction of the intact passenger ejection (Figures 6D and S10D), which supports the model that dissociation of the 3’ half of the guide strand from the passenger strand by the PAZ domain makes the post-seed guide region accessible to target RNAs. The TAPE mechanism also explains why AGO2 could autonomously eject the −4-nt overhanging end passenger strand (Figure 2I): in ej-RISC, this passenger strand cannot maintain pairing with the guide seed region, and, therefore, it can be released even without the aid of target RNA once the PAZ domain captures the guide 3’ end to trigger the wedg-to-ej transition. Our ej-RISC structure and functional study could explain the previous report that seed mismatches facilitate unwinding.^51^ Altogether, the ej-RISC structure represents a state just before AGO2 fully embraces the 5’ monophosphate group of the guide, which is a critical step in completing RISC assembly.

### TAPE operates for cleaved passengers

Early studies reported that passenger-strand cleavage is essential when fly Ago2 and human AGO2 eject the siRNA passenger strand.^40,41,51,67,68^ In addition, using the human HeLa S100 extraction, the Zamore group reported that human AGO2 forms mat-RISC even when incubated at 30°C with an siRNA-like miR-1 duplex, whose passenger contains a phosphorothioate linker between p10 and p11 and thus was not cleaved.^41^ The Martinez group also observed this bypass mechanism, in which the intact passenger is ejected.^68^ Later, the Shin group’s dedicated study on the bypass mechanism reported that, in cell lysate, human AGO2 can eject the passenger strand more efficiently, in a slicer-independent manner, at 0.75 mM Mg^2+^ and 37°C, rather than at 3.75 mM Mg^2+^ and 25°C.^19^ To reconcile these findings, we performed a passenger-ejection assay in the presence of 5 mM Mg^2+^ at 37°C. Under the conditions, AGO2 cleaved 60.2% and 51.8% of the passenger strands in the absence and presence of target RNAs, respectively (Figures 6E and S10E), which indicated that the existence of target RNAs barely influences AGO2-mediated passenger cleavage, if at all. In striking contrast, the addition of target RNAs markedly enhanced the ejection of a cleaved passenger strand from 0.4% to 24.0% (60-fold) (Figure 6E). These results suggest that TAPE operates not only during slicer-independent passenger ejection but also contributes substantially to slicer-dependent ejection.

### Mg^2+^ stabilizes passenger-cleavage RISC state

To test whether slicer-independent passenger dissociation could be favored in the presence of Mg^2+^ when passenger cleavage is suppressed, we examined an siRNA duplex containing a phosphorothioate linkage between p10 and p11, which is a modification that strongly reduces passenger cleavage by catalytically active AGOs.^41^ As expected, even in the presence of Mg^2+^, only 5.4% of the modified passenger strand was cleaved, but the addition of target RNAs resulted in inefficient release of the intact modified passenger strand, which is comparable to that observed for the unmodified duplex (Figure 6F, top, and S10F). In the absence of Mg^2+^, the intact modified passenger strand was released with much higher efficiency, which is similar to the unmodified passenger (Figure 6F, bottom, and S10F). These results indicate that incorporation of a phosphorothioate linkage at the scissile phosphate is not sufficient to bias AGO2 toward slicer-independent passenger dissociation under Mg^2+^-containing conditions. Instead, the data suggest a model in which Mg^2+^ stabilizes a passenger-cleavage-competent RISC (pc-RISC) conformation, thereby disfavoring conformational change to ej-RISC, which is a state required for release of an intact passenger strand.

### All human AGOs need TAPE for RISC maturation *in vitro*

A previous study reported that naturally slicer-deficient human AGO1 and AGO4, as well as catalytically active AGO3, unwind siRNA duplexes in the HEK293T cell lysate at 37°C but not at 25°C.^19^ Our study revealed that TAPE operated for human AGO2 without passenger cleavage (Figures 6A–6C), which prompted us to test whether other human AGOs loaded with siRNA duplexes undergo TAPE in the absence of Mg^2+^. After incubation with the siRNA-like duplex of miR-20a, FLAG-AGO1, -AGO3, and -AGO4 remained stably associated with the passenger strand at 37 °C, and the addition of target RNAs initiated TAPE (Figures 6G and S10G). This result indicates that all four human AGOs are subject to TAPE.

## DISCUSSION

**Figure 7.**
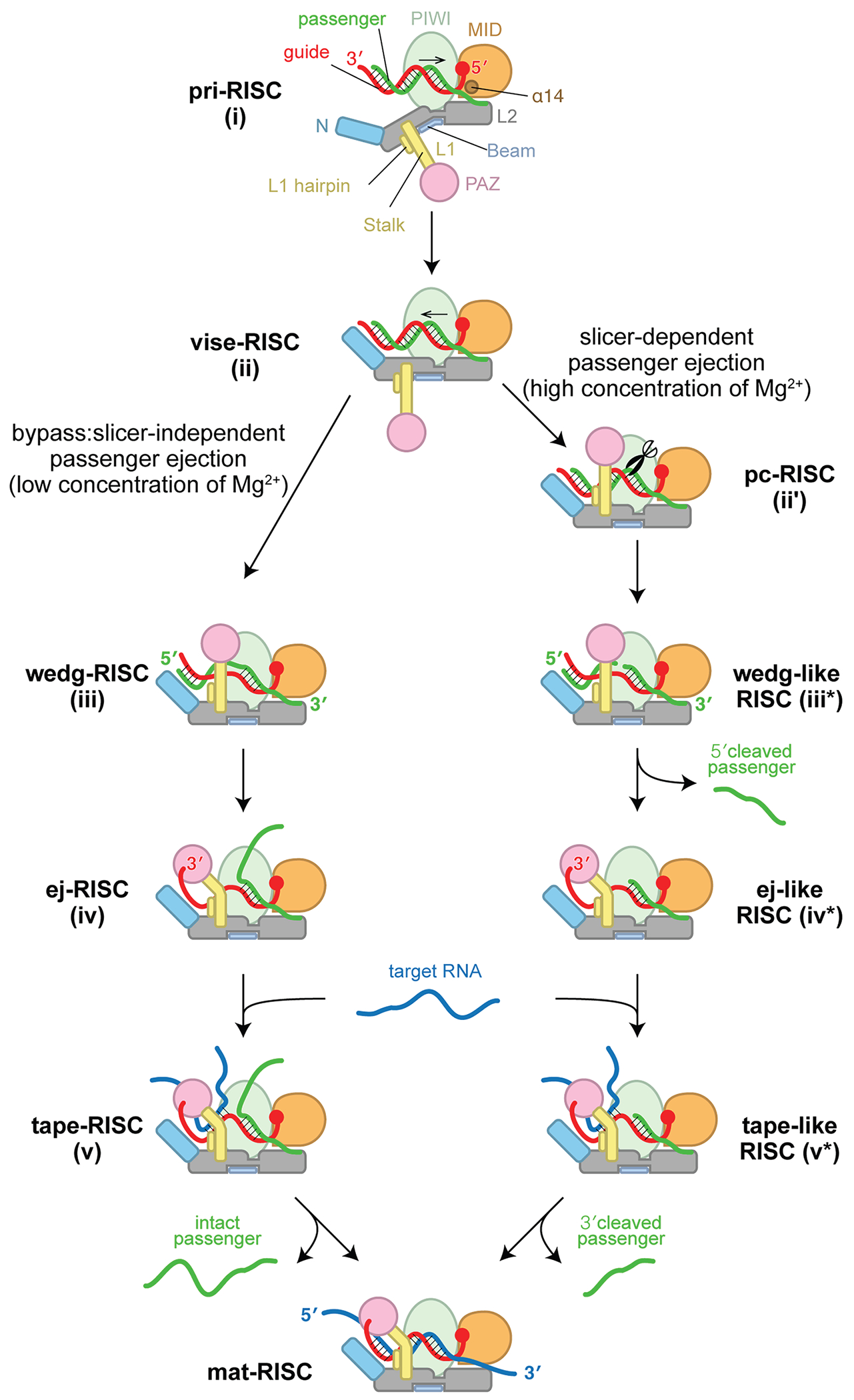
Model of RISC assembly of human AGO2 RISC assembly of AGO1-4 proceeds through at least five states, pri-, vise-, wedg-, ej-, and tape-RISCs in the slicer-independent pathway AGO2 follows similar intermediates but additionally forms pc-RISC (state ii’) during slicer-dependent passenger ejection (right pathway). States with ’ or * are inferred from cryo-EM structures and biochemical assays.

Our study revealed multiple pre-RISC intermediates that were associated with distinct conformational rearrangements (Figure 7). Because cryo-EM captures ensembles rather than time-resolved trajectories, the temporal order of these states cannot be directly inferred. Nevertheless, we propose the sequence pri → vise → wedg → ej → tape → mat as a plausible on-pathway progression based on structural continuity, functional consistency, and temperature-dependent population shifts. Structurally, this ordering follows the physiological trajectory of RISC assembly: duplex loading, progressive duplex engagement, unwinding, passenger ejection, and maturation into the bilobed mat-RISC. In pri-RISC, AGO2 binds only one end of the duplex and uniquely positions α14 between the guide 5’ end and passenger 3’ end, which is consistent with strand selection. Vise-RISC introduces engagement of the distal duplex end by the lysine- and arginine-rich N-domain loop, which is supported by mutational defects in RISC maturation. In wedg-RISC, the L1 hairpin and Stalk begin to distort the duplex, and disease-associated mutations in these elements impair progression to mat-RISC, which places this state downstream of pri- and vise-RISCs. Transition to ej-RISC is marked by PAZ engagement of the guide 3’ end and partial duplex unwinding that prevents reannealing of the passenger strand; disruption of PAZ anchoring similarly blocks maturation. Finally, ej-RISC retains seed pairing while exposing the post-seed region, enabling TAPE, which drives conversion to mat-RISC. This sequence is further supported by increasing structural completeness: as assembly proceeds (pri → vise → wedg → ej → mat), additional domains become ordered and coordinately engaged, which culminates in the bilobed architecture characteristic of mat-RISC. Temperature-dependent distributions provide orthogonal support. Because conformational transition rates scale approximately as exp(−ΔG^‡^/RT), where ΔG^‡^ is the activation free energy barrier, R is the gas constant, and T is the absolute temperature, lower temperatures slow transitions and kinetically trap earlier intermediates, whereas higher temperatures allow crossing larger activation barriers. Consistent with this principle, pri-RISC was observed at both 4°C and 21°C, which suggested that it represents a thermodynamic minimum, whereas vise- and wedg-RISCs were enriched at 4°C, and ej- and mat-RISCs appeared only at 21°C. The co-occurrence of ej- and mat-RISCs further supports the interpretation that ej-RISC precedes maturation. Together, these structural and functional observations argue that the captured conformations represent a coherent series of on-pathway intermediates rather than unrelated off-pathway states or artifacts. We note, however, that alternative pathways cannot be fully excluded without time-resolved approaches, and thus our nomenclature (pri-, vise-, wedg-, ej-, and tape-RISC) reflects mechanistic assignments rather than definitive kinetic ordering.

Because divalent cations were depleted from our cryo-EM samples, the pri-, vise-, wedg-, and ej-RISC structures represent intermediates of the slicer-independent assembly pathway (“bypass”) (Figure 7, left).^41,68^ In pri-RISC, AGO2 binds only one end of the duplex, which allows indiscriminate loading of siRNA and miRNA duplexes, and thus is consistent with previous reports.^50,51^ AGO2 then transitions to vise-RISC, which appears to accommodate both duplex types, and suggests that both slicer-dependent and slicer-independent pathways pass through pri- and vise-RISC (Figure 7). When AGO2 loads an siRNA duplex, the L1 hairpin and Stalk interact with the central region of the duplex but do not efficiently unwind it, which prevents the capture of the guide 3’ end by the PAZ domain. Instead, these interactions likely reposition the duplex toward the catalytic tetrad, DEDH (Asp-Glu-Asp-His), of the PIWI domain.^69^ Eukaryotic AGOs coordinate two divalent metal ions via conserved catalytic aspartates to anchor the scissile phosphate of the target strand.^70^ Similarly, the passenger scissile site of the relocated duplex may be anchored by Mg^2+^ ions, which stabilizes the pc-RISC conformation and promotes entry into the slicer-dependent pathway (Figure 7, right).^70^ Consistent with this model, AGO2 inefficiently ejected a passenger strand containing a phosphorothioate linkage between p10 and p11 in the presence of Mg^2+^ (Figure 6F), which suggests that Mg^2+^-dependent anchoring stabilizes the duplex and prevents passenger release without cleavage. AGO2 would therefore stall in pc-RISC. This mechanism may explain why passenger ejection by AGO2 often proceeds through the slicer-dependent pathway. Without divalent cations, however, the duplex is not anchored and is instead partially unwound by the L1 hairpin and Stalk, as observed in wedg-RISC. Our assays further showed that AGO2 can eject intact passenger strands even in the presence of Mg^2+^ (Figure 6E), which indicated that both slicer-dependent and slicer-independent ejection pathways operate, and thus is consistent with earlier studies.^41,68^ Following passenger cleavage, the PAZ domain likely captures the guide 3’ end to promote dissociation of the 5’ cleavage fragment (Figure 7, right). Supporting this model, efficient ejection of the 3’ cleavage fragment requires TAPE, which depends on accessibility of the guide postseed region after release of the 5’ passenger fragment (Figures 6C and 6E). This configuration mirrors the ej-RISC structure, in which the seed region remains blocked while the post-seed region becomes accessible. We therefore infer that the slicer-dependent pathway proceeds through conformations analogous to wedg-, ej-, and tape-RISCs—termed wedg-like, ej-like, and tape-like RISCs—which differ primarily by the additional passenger-cleavage state pc-RISC (state ii’) (Figure 7, right). Because a 2-nt 3’ overhanging end at the guide end promotes PAZ binding and efficient silencing,^12,48,71^ the duplex likely collapses primarily in the central region, while preserving the terminal duplex segment recognized by the PAZ domain.

How do other human AGO paralogs form mat-RISC? Although AGO3 can cleave RNAs,^72^ its slicing activity is efficient only when programmed with 14–15-nt cleavage-inducing tiny RNAs (cityRNAs).^73,74^ In addition, AGO3 shortens its nucleic acid-binding channel with an AGO3-specific insertion (3SI),^72^ thus preventing the accommodation of ~22-nt siRNA duplexes and passenger cleavage.^75–77^ AGO1 lacks catalytic activity because the catalytic histidine is replaced with arginine, yet it retains two catalytic aspartates capable of coordinating divalent metal ions. This might explain why AGO1 slows RISC maturation at high Mg^2+^ concentrations when loaded with siRNA. A conserved segment 7 (cS7) of AGO1 protrudes into the nucleic acid-binding channel^78^ and likely restricts duplex access to the pseudo-catalytic site. AGO4 replaces one catalytic aspartate with glycine and contains an AGO4-specific insertion (4SI) within the channel.^46^ These structural features likely prevent AGO1, AGO3, and AGO4 from stalling in a pc-RISC-like conformation at physiological Mg^2+^ concentrations. Consequently, regardless of the loaded duplex types, these paralogs likely form mat-RISC exclusively through the slicer-independent pathway (Figure 7, left). In this pathway, the PAZ domain captures the guide 3’ end to promote passenger ejection, as observed during the wedg- to ej-RISC transition in the current study. Consistent with this, deletion of the PAZ domain abolishes passenger ejection and RNAi activity in these AGOs.^79^

For two decades, mRNAs have been viewed primarily as targets of miRNAs and siRNAs,^20–28^ except in cases of TDMD, where specific mRNAs trigger miRNA decay.^29–35^ In both cases, mRNAs interact with mat-RISCs. Our results instead suggest that mRNAs can also bind pre-RISCs and facilitate passenger removal during RISC maturation. Targets pairing with only a few nucleotides of the guide promote modest but measurable passenger ejection (Figures 6A and 6B), which indicates that TAPE does not require full complementarity. Because cellular mRNAs collectively contain numerous partially complementary sites for many miRNAs, such interactions may collectively drive TAPE in cells. This model could explain why transfected siRNA duplexes lacking perfectly complementary targets still assemble into mat-RISCs in endogenous AGOs. Although our minimal *in vitro* system clearly demonstrates the contribution of TAPE to RISC assembly, it likely functions in cells as one of several factors that enhance passenger ejection rather than as an absolute requirement.

The TAPE mechanism also provides a molecular explanation for target-mediated miRNA protection (TMMP). Großhans and coworkers showed in *C. elegans* that target availability increases the level of AGO-associated miRNAs,^38^ and that target RNAs can promote the ejection of the opposing strand during RISC assembly. Similar observations were later reported in human systems,^39^ where limited target abundance results in inefficient passenger ejection despite duplex loading into AGO2. These findings indicate that target abundance stabilizes miRNAs and their cognate RISCs, although the mechanism has remained unclear. The TAPE model proposed here provides a plausible explanation: target RNAs promote formation of cognate RISCs and thereby protect loaded miRNAs from degradation. Thus, abundant cellular mRNAs may actively participate in RISC assembly and indirectly regulate their own expression once a miRNA is expressed. This mechanism further suggests that TAPE could contribute to the co-evolution of miRNAs and their targets.

### Limitations of the study

Our cryo-EM datasets capture multiple AGO2-RNA conformations that we interpret as intermediates of RISC assembly. However, because these structures were obtained from equilibrium samples rather than time-resolved experiments, the data cannot formally establish the order of transitions or exclude the possibility that some conformations represent off-pathway states. Also, our bead-based passenger-ejection assays monitor released passenger strands in the supernatant fraction and therefore cannot formally exclude the possibility that transient passenger release is followed by rapid rebinding before detection. Although several observations suggest that such events are unlikely to dominate under our experimental conditions (Figure 6), the current experimental design cannot completely rule out short-lived rebinding events.

## RESOURCE AVAILABILITY

### Lead contact

Further information and requests for resources and reagents should be directed to the lead contact, Kotaro Nakanishi (nakanishi.9@osu.edu).

### Materials availability

Unique reagents generated in this study are available from the lead contact upon request with a completed material transfer agreement.

## STAR★METHODS

### EXPERIMENTAL MODEL AND STUDY PARTICIPANT DETAILS

HEK293T cells were obtained from ATCC [CRL-1573] and cultured in Dulbecco’s modified Eagle’s medium (DMEM) (Gibco) supplemented with 10% FBS (Gibco) at 37 °C and 5% CO_2_. Sf9 cells grown in ESF 921 media at 28 °C and 120 rpm were used to make viruses for AGO protein expression. *T. ni* cells grown in ESF 921 media at 28 °C and 120 rpm were used to express AGO proteins. DH10bac *E.coli* were grown on LB Agar at 37 °C for transformation of pFastBac^™^HTB, and in LB Broth at 37 °C and 200 rpm for bac-mid amplification. DH5α *E. coli* were grown on LB Agar plates at 37 °C for cloning and plasmid amplification. Rosetta 2(DE3)pLysS *E. coli* were grown in LB media at 37 °C and 200 rpm for maintenance and at 37°C for 5 hours for ISG20 overexpression.

### METHOD DETAILS

#### Pre-RISC purification

Human AGO2 was expressed in *T. ni* cells using the Bac-to-Bac Baculovirus Expression Systems (Gibco^™^). *T. ni* cells from 2-3 L culture were resuspended in Buffer A (50 mM Tris-HCl pH 8.0, 300 mM NaCl, 0.5 mM TCEP) supplemented with 1 mM PMSF and SigmaFAST Protease Inhibitor Cocktail, EDTA-free (Sigma). Cells were lysed in a C3 Homogenizer at 4 °C, followed by centrifugation at 23,000 rpm. The supernatant was added to 10-20 mL Ni Sepharose HP resin (Cytiva) pre-equilibrated in Buffer A. The mixture of beads and supernatant was incubated on a shaker at 4 °C and 100 rpm for 1 hour. The beads were then recovered by centrifugation at 4,000 x *g* for 3 min. Beads were washed 4 times with 4 column volume (CV) Buffer A and twice with 4 CV Buffer B (50 mM Tris-HCl pH 8.0, 300 mM NaCl, 25 mM imidazole, 0.5 mM TCEP). The beads were resuspended in 2 CV Buffer B with 5 mM CaCl_2_, after which 10 μL micrococcus nuclease (Takara) per liter of original cell culture was added. Digestion was performed at RT for 1 hour, followed by 6 washes of 4 CV Buffer B. The beads were loaded onto a gravity column and AGO2 protein was eluted with Buffer C (50 mM Tris-HCl pH 8.0, 300 mM NaCl, 300 mM imidazole, 0.5 mM TCEP). Sample was dialyzed overnight at 4°C in the presence of TEV to cleave the His-tag. AGO2 protein was subjected to 2 × 5 mL HisTrap HP (Cytiva) columns to remove the cleaved His-tag and non-cleaved AGO2. AGO2 protein from the flow-through of the second nickel column was estimated by SDS-PAGE using known concentrations of bovine serum albumin (BSA). The siRNA-like duplex of miR-20a [RNA:AGO2=1:2] was added to AGO2 from the second Ni column for the loading. 23-nt miR-20a guide was annealed with 1.1x 5’-biotylated passenger by heating at 90 °C for 2 min, followed by incubation at room temperature for 10 min and storage on ice before addition. The AGO2 and siRNA-like duplex mixture was incubated overnight at 4 °C to form the pre-RISC. 2x 1 mL HiTrap Q FF columns (Cytiva) were then used to remove free RNA. ~400 μL monomeric avidin beads were added into the flow-through of HiTrap Q FF after 3 washes with 1 mL 1x PBS. The mixture was subsequently incubated on a rotor at room temperature (21 °C sample) for 1 hour or at 4 °C (4 °C sample) for 3 hours. All samples were then loaded onto a gravity column and washed with ~20 mL HEPES buffer (20 mM HEPES pH 7.5, 150 mM NaCl, 0.5 mM TCEP). The bound pre-RISC was eluted with ~5 mL ~11 mM biotin in HEPES buffer on ice. The eluted sample was dialyzed overnight at 4 °C against 100 mL HEPES buffer, followed by a second dialysis against fresh HEPES buffer for 2 hours to remove biotin. The dialyzed sample was concentrated down to ~100 μL and then loaded onto a Superdex 200 increase 10/300 GL column (Cytiva) at 4 °C. The fractions containing the protein/RNA complex were pooled and concentrated. The concentration of pre-RISC was measured by A280 using a Biospectrometer (Eppendorf). 8 M urea 20% (29:1) acrylamide/bis-acrylamide denaturing gels were used to check the integrity of both the guide and passenger RNAs. SDS-PAGE gels were used to check the purity of AGO2. The concentrated samples were used to make cryo-EM grids and/or flash-frozen and stored at −80 °C.

#### Purification of homogeneous AGO2 RISC

Homogeneous AGO2 loaded with 23-nt miR-20a was purified as described previously,^74^ using the ARPON method.^84^ *T. ni* cells from 3 L culture expressing AGO2 were lysed, and AGO2 protein was purified with Ni Sepharose HP and HiTrap Q FF columns as indicated above. The amount of eluted AGO2 protein was estimated by SDS-PAGE using known concentrations of BSA after both the first and second Nickel columns. After the first nickel column, guide RNA was added to samples containing AGO2 protein [RNA:AGO2=1:2] and incubated on ice for 15 min prior to dialysis in the presence of TEV. After the second nickel column, pre-equilibrated NeutrAvidin beads (Thermo Scientific^™^) were mixed with capture oligo and pre-incubated in Buffer D (50 mM Tris-HCl pH 8.0, 300 mM NaCl, 0.01% CHAPS, 2 mM Mg(OAc)_2_, 0.5 mM TCEP) for 1 hour at 4°C, then washed with Buffer D once to remove unbound capture oligo. The NeutrAvidin beads with bound capture oligo were added to RISC-containing samples [capture oligo:AGO2=1:10] and incubated at RT for 1.5 hours before transferring to a column to wash the beads with 5 x 4 CV Buffer E (30 mM Tris-HCl pH 8.0, 100 mM KOAc, 0.01% CHAPS, 2 mM Mg(OAc)_2_, 0.5 mM TCEP) and 5 x 4 CV Buffer F (30 mM Tris-HCl pH 8.0, 2 M KOAc, 0.01% CHAPS, 2 mM Mg (OAc)_2_, 0.5 mM TCEP). Beads were resuspended in 3 CV of Buffer G (30 mM Tris-HCl pH 8.0, 1 M KOAc, 0.01% CHAPS, 2 mM Mg (OAc)_2_, 0.5 mM TCEP) and elution oligo was added [elution oligo:capture oligo=3:1]. The mixture was incubated at RT for 2 hours, after which the beads were washed with 2 x 3 CV of storage buffer (50 mM HEPES pH 7.5, 150 mM NaCl, 0.5 mM TCEP) and dialyzed against the storage buffer at 4°C overnight. Elution oligo was removed by running the RISC sample through a third HiTrap Q FF column. Flow-through was concentrated and loaded to a Superdex 200 increase 10/300 GL column (Cytiva) with storage buffer. AGO2 purity of the RISC was evaluated by SDS-PAGE and the guide RNA purity resolved on a denaturing gel. RISC concentrations were measured by A280. The RISC was directly frozen in liquid nitrogen and then stored at −80°C.

#### Cryo-EM sample preparation and data acquisition

For the 4°C sample, 0.31 μL of 50 mM BS3 (bis(sulfosuccinimidyl)suberate) (final 2 mM) was added into 6.6 μL 0.44 mg/mL pre-RISC, followed by incubation on ice for 1 hour. 0.77 μL of 200 mM Tris-HCl pH 7.5 was added to stop the crosslinking. The final concentration of the pre-RISC sample was 0.38 mg/mL. For the 21°C sample, 1 μL of 10 mM BS3 (final 1 mM) was added to 9 μL of 0.49 mg/mL pre-RISC. The sample was then incubated on ice for 1 hour. 1 μL of 200 mM Tris-HCl, pH 7.5, was added to stop the crosslinking reaction, followed by the addition of 1 μL of H_2_O to make the final volume 12 μL. The final concentration of the pre-RISC sample was 0.37 mg/mL. 3 μL of the crosslinked sample was applied to one glow-discharged Quantifoil R1.2/1.3 300 mesh UltraAuFoil grid (Electron Microscopy Sciences) at 4°C using an FEI Vitrobot Mark IV (Thermo Scientific^™^). All grids were blotted for 4 seconds with 100% humidity and blot force 1 and then vitrified in liquid ethane. The grids were screened using a Thermo Scientific^™^ Glacios microscope at the Center for Electron Microscopy and Analysis (CEMAS) at The Ohio State University (OSU).

AGO2:23-nt miR-20a (0.35 mg/mL) in storage buffer was incubated with the 18-nt target (1.2x) on ice for 5 min in the presence of 5 mM MgCl_2_, followed by incubation with 2 mM BS3 on ice for 30 min. Reactions were quenched with addition of 50 mM Tris-HCl pH 7.5 for a minimum of 15 minutes on ice. Grids were made and screened as described above.

A total of 10,091 micrographs were collected for the 4°C pre-RISC sample using a 300-kV Titan Krios microscope (Thermo Fisher Scientific) equipped with a K3 direct electron detector in super-resolution mode, with magnification of 81,000x, a pixel size of 0.426 Å, a defocus range from −0.6 to −1.6 μm, and a total dose of 50 e^−^ Å^−2^ over 50 fractions.

A total of 11,296 micrographs were collected for the 21°C pre-RISC sample using a 300-kV Titan Krios microscope (Thermo Scientific^™^) equipped with a K3 camera in counting mode, with magnification of 105,000x, a pixel size of 0.835 Å, a defocus range from −0.8 to −2.4 μm, and a total dose of 50 e^−^ Å^−2^ over 40 fractions.

A total of 8,149 micrographs were collected for the AGO2:23-nt miR-20a:18-nt target using a Titan Krios microscope (Thermo Scientific^™^) equipped with a Gatan K3 camera in counting mode, with magnification of 105,000 x, a pixel size of 0.7296 Å, a defocus range from −0.8 to −2.2 μm, and a total dose of 50 e^−^ Å^−2^ over 50 fractions.

#### Cryo-EM data processing, model building and refinement

Cryo-EM data processing workflows for pre-RISC samples are shown in Figures S2 and S6, respectively. Both datasets were processed with cryoSPARC.^80^

For the 4°C pre-RISC sample, 6,164 micrographs from the first data set were imported into cryoSPARC for patch motion correction and patch CTF (contrast transfer function). After curation, 5,922 micrographs were used for the following processing. Blob picker with particle diameters range 50-120 Å was used to pick 1,251,530 particles from 1,000 micrographs. Those particles were extracted with box size 256 pixels and 2x binning, followed by 1 round of 2D classification with 50 classes. 3 classes with 110.548 particles were selected for Topaz training on all 5,922 micrographs, followed by Topaz extract. After manually inspecting, 1,719,698 particles were extracted with box size 256 pixels and 2x binning. After 2 rounds of 2D classification, 752,547 particles were used for *ab-initio* reconstruction with 5 classes. A second data set for the 4°C pre-RISC sample with 3,927 micrographs was processed similarly, resulting in 990,900 good particles. Good particles from data set 1 and 2 were combined for the following processing. The two maps with clear features of RNA duplex from the above heterogeneous refinement were used for heterogeneous refinement with 6 junk reference maps, which were generated by an *ab-initio* job with 3 classes using 100 particles with each class used twice. A total of 3 rounds of heterogeneous refinement were performed using the best 2 maps from the previous round and the same 6 junk reference maps.

For the class corresponding to pri-RISC, the 237,554 particles were re-extracted with box size 256 pixels without binning and then reference motion corrected. One round of 2D classification was used to further remove bad particles. The selected 203,008 good particles were used for non-uniform (NU) refinement, followed by a local refinement with a full mask. The final resolution of the pri-RISC map is 3.79 Å based on Fourier Shell Correlation (FSC) at 0.143. The map was sharpened for docking and modeling purposes.

For the class corresponding to vise- and wedg-RISC, the 205,047 particles were re-extracted with box size 256 pixels without binning and then reference motion corrected. An *ab-initio* reconstruction followed by a heterogeneous refinement with 2 classes were performed, leading to further removal of 43,312 particles. The remaining 161,301 particles were used for a 3D classification with 2 classes. Each of the 2 classes were further processed by an *ab-initio* reconstruction and a heterogeneous refinement with each having 2 classes. The 2 better classes with 64,361 and 64,344 particles were processed by NU and local refinements, leading to 3.87 and 3.77 Å maps corresponding to vise- and wedg-RISC, respectively. The maps were sharpened for model building purpose.

For the 21°C pre-RISC sample, 11,296 micrographs were imported for patch motion correction and CTF. After curation, 11,165 micrographs were used for further processing. Blob picker with particle diameters range 50-120 Å was used to pick 454,870 particles from 1000 micrographs. Those particles were extracted with box size 276 pixels, followed by 1 round of 2D classification with 50 classes. One class with 11,761 particles were selected for Topaz training on all 11,165 micrographs, followed by Topaz extract, particle extraction, and 1 round of 2D classification. Two more rounds of Topaz training, extract, particle extraction and 2D classification led to 3,303,317 particles, which were used for *ab-initio* reconstruction with 7 classes, followed by 1 round of heterogeneous refinement using the same 7 classes. Two more rounds of heterogeneous refinement using the 7 classes and 3 junk reference volumes were performed, generating the best map with 133,392 particles. The 3 junk reference volumes were generated the same way as above. An *ab-initio* reconstruction and heterogeneous refinement with 2 classes using the 133,392 particles were carried out, leading to 2 different maps with particle number 63,912 and 66,044, respectively. The particles of the two classes were reference motion corrected, followed by NU refinement and local refinement with whole masks. The final resolutions of the two maps are 3.45 and 3.28 Å based on FSC at 0.143, corresponding to mature and ej-RISCs, respectively.

For the structure of AGO2 in complex with 23-nt miR-20a and the 18-nt target, 8,149 movies were imported into CryoSPARC. After patch motion correction and CTF, 8,031 movies were selected for further process. Blob picker identified 3,590,156 particles in total, which were filtered for potential non-particle noise to 3,096,063 particles for extraction with a box size of 488x488 pixels (Fourier cropping reduced to 244x244). 2D classification revealed that 3 classes displayed an ideal side orientation of the protein, which exposes the major domains of AGO2 alongside the RNA binding channel for a total of 205,866 particles. The selected 3 classes were used to train a Topaz model. The resulting model was exposed to the 8,031 exposures to yield a total of 842,078 extracted particles. Back-to-back heterogenous refinements across 5 classes resulted in one predominant class (330,801 particles) that displayed all main AGO domains. This class was enhanced by an NU- and then global CTF- refinement that produced a map with 2.85 Å resolution. Afterwards, to improve map quality in blurry regions, a final NU- and local- refinement were utilized to produce a map at 3.01 Å, determined by the gold-standard FSC criterion of 0.143. Data processing workflows is shown in Figure S8A.

An AlphaFold3 predicted structure consisting of human AGO2 T406-A859 and the 23-nt miR-20a duplex sequences was used as the initial model of pri-RISC. An AlphaFold3 structure consisting of full-length human AGO2 and the same duplex was used as the initial model of vise- and wedg-RISCs. An AlphaFold3 structure consisting of full-length human AGO2, the 23-nt miR-20a, and passenger p1-p9 with 2-nt 3′ overhang was used as the initial model of ej-RISC and mat-RISC and PDB:4W5N was used as another initial model for mat-RISC. Docking of initial models to cryo-EM maps were done in ChimeraX. The models were then manually adjusted in Coot, refined in ChimeraX ISOLDE and Phenix. AGO2 PDB:4W5N and 4W5O were used as the initial reference models for the AGO2-RISC structure and aligned in UCSF ChimeraX. Backbone atoms were manually fit to the EM map alongside iterative real-space refinement in Phenix. The guide and target strands were built by aligning the WT MID domain from AGO2 bound to a target RNA (PDB:4OLA) with the MID domain from the new structure. The final position of the RNA was adjusted with real space refinement in Coot and Phenix.

##### Recombinant protein purification

Recombinant FLAG-AGO2-WT, -F2L3, −ΔF182, and L192P, as well as FLAG-AGO1, -AGO3, and -AGO4 were purified as previously reported.^72^ The proteins were expressed in *T.ni* cells as stated above and homogenized in Buffer A′ (1x PBS, 500 mM NaCl, 40 mM imidazole, 10 mM β-ME) supplemented with 1 mM PMSF and SigmaFAST Protease Inhibitor Cocktail, EDTA-free (Sigma). The supernatant was loaded onto Ni Sepharose HP resin (Cytiva), washed with Buffer A’, and eluted with a linear gradient to 50% Buffer B’ (1x PBS, 500 mM NaCl, 1.5 M imidazole, 10 mM β-ME). The sample was dialyzed overnight with TEV protease against Buffer C’ (1x PBS, 500 mM NaCl, 10 mM β-ME), and the cleaved His-tag was removed by loading the sample onto a 5 mL Ni Sepharose HP column (Cytiva).The flow-through sample was dialyzed against Buffer D’ (10 mM Tris-HCl pH 8.0, 80 mM KCl, 10 mM β-ME) and then loaded onto Mono Q 5/50 GL (Cytiva) equilibrated with Buffer E’ (10 mM Tris-HCl pH 8.0, 50 mM KCl, 10 mM β-ME). The flow-through sample was collected and dialyzed against Buffer F’ (20 mM Tris-HCl pH 7.5, 300 mM NaCl, 10 mM β-ME), followed by ultrafiltration. The concentrated sample was loaded onto a HiLoad 16/600 Superdex 200 column (Cytiva) in Buffer G’ (20 mM Tris-HCl pH 7.5, 300 mM NaCl, 5 mM DTT). After concentration, the purified protein was stored at − 80°C.

His-SUMO-ISG20 was purified as previously described.^42^ The protein was expressed in Rosetta 2(DE3)pLysS *E. coli* cells which were homogenized in Buffer A’ with 5% glycerol and supplemented with 1 mM PMSF. The supernatant was loaded onto a 5 mL HisTrap HP Column (Cytiva), which was then washed with Buffer A’, and eluted with a stepwise gradient to 100% Buffer B’ including 5% Glycerol. The sample was dialyzed overnight against 20 mM Tris-HCl pH 7.5, 300 mM NaCl, and 10 mM β-ME, and concentrated by ultrafiltration and flash-frozen in liquid nitrogen prior to storage at −80°C.

##### RNA duplex annealing

5’-P guide RNA was annealed with 1.2x 5’-OH passenger strand by incubating RNA on a 90°C heat block in 1x trimming buffer (25 mM HEPES pH 7.5, 50 mM KCl, 5 mM DTT, 0.2 mM EDTA) for 2 min, followed by moving the 90°C heat block to room temperature to allow the sample to slow cool to < 30°C. Formation of RNA duplex was confirmed by running a 16% native polyacrylamide gel in 0.5x TBE at 4°C.

##### In vitro guide RNA trimming assay

Guide RNAs were radiolabeled using *γ*-^32^P ATP (3,000 Ci mmol^−1^; Revvity) with T4 Polynucleotide kinase (Thermo Scientific^™^) at 37°C for 1.5 hours, followed by inactivation of the kinase at 90°C for 2 min. Unincorporated *γ*-^32^P ATP was removed using MicroSpin^™^ G-25 columns (Cytiva). 2.5 μM 5’-P guide RNA spiked with 5’-^32^P guide was annealed with 1.2x 5’-OH passenger strand as described above.

0.5 μM small RNA duplex was incubated for 1 hour at 25°C with 7.5 μM recombinant FLAG-AGO2 in 1x trimming buffer supplemented with 0.1 mg/mL BSA. Pre-equilibrated anti-FLAG^®^ M2 Magnetic Beads were added into the mixture at the same temperature and incubated for 1 hour to allow binding of protein-RNA complex to the beads. The beads were then washed 8 times with 10 bead volumes of wash buffer (50 mM Tris-HCl pH 7.5, 300 mM NaCl, 0.05% NP-40) using a magnetic stand, followed by 3 washes with 10 bead volumes of 1x PBS to remove NP-40. The beads were subsequently washed with 2 bead volumes of 1x trimming buffer once. 100 μL of 1x trimming buffer with 2 mM MnCl_2_ was added to each sample. Each sample was split into 2 halves, with one half of each sample saved as ‘no-ISG20’ control, and 500 pmol His-SUMO-ISG20 was added to the other half for trimming. All samples were incubated at 25°C, shaking at 1,400 rpm for 4 hours. The beads were washed 4 times with wash buffer, followed by quenching with 20 μL of 2x quenching buffer (8 M urea, 1 mM EDTA, 0.05% (w/v) xylene cyanol, 0.05% (w/v) bromophenol blue, 10% (v/v) phenol, 20% glycerol) and heating at 90°C for 3 min. Loaded all supernatant onto a 20% denaturing gel. Phosphor images were taken by a Typhoon Imager (GE Healthcare), and band intensity was quantified using Image Lab (Bio-Rad). The results were graphed using GraphPad Prism (GraphPad Software, Inc.)

Naked RNA trimming was performed by incubating 0.1 μM single-stranded 5’-^32^P guide or RNA duplex with 0.5 μM His-SUMO-ISG20 in 20 μL of reaction at 25°C. 4.5 μL of samples were quenched at 2, 5, 10, and 20 min. A 0-minute sample was prepared by adding RNA directly into the 2x quenching buffer. Trimming samples were loaded on 20% denaturing gels. Phosphor images were taken by Typhoon Imager (GE Healthcare) and band intensity was quantified using Image Lab (Bio-Rad). The results were graphed using GraphPad Prism (GraphPad Software, Inc.)

##### Cloning

pCAGEN FLAG-AGO2-Δα14 was created from the wild-type construct with site-directed mutagenesis, using PCR primers designed to anneal to the mutation sites. A DNA fragment comprising FLAG tag sequence at the 5’ end of an AGO2 sequence, of which L356-T368 of the alpha helix 7 is replaced with 5 glycines, flanked by EcoR I and Not I restriction sites at the 5’ and 3’ end, respectively, was ordered for GenScript. A similar DNA fragment was ordered from GenScript for the AGO2 N domain mutant (K62A, K65A, R68A, R69A). The DNAs were digested with EcoR I and Not I and ligated to digested and dephosphorylated pCAGEN vector. The ligation product was transformed into DH5α competent cells. Plasmids were isolated. All plasmids were verified by Sanger sequencing (OSUCCC Shared Resources) or PLASMIDSAURUS (https://plasmidsaurus.com).

##### Transfection

HEK293T cells were cultured in 10 cm plates. At 60-70% confluency, the old media were replaced with fresh media and the cells were transfected with 10 μg of pCAGEN encoding FLAG-AGO2-WT or mutant using 45 μL of TransIT-X2 and 1.5 mL OMEM. After the transfection, the plate was incubated in a 37°C incubator with 5% CO_2_. After 48 hours, the growth media were removed, and the cells were washed twice in 1x PBS prior to centrifugation at 2,000 x g for 5 min at 4°C. The supernatant was removed and the cell pellets were flash-frozen in liquid nitrogen and stored at − 80°C.

##### Western Blot

HEK293T cells from transfection were lysed using 1× RIPA buffer (Cell Signaling Technology) containing 1 mM PMSF. 280 μg of the whole cell lysate was resolved on Bolt^™^ 4-12% Bis-Tris gels (Invitrogen^™^) and transferred onto nitrocellulose membranes. The membranes were incubated with primary anti-FLAG antibody (1:1,000, Genscript) and anti-alpha-tubulin antibody (1:1,000, Cell Signaling Technology), and secondary goat anti-mouse (1:15,000, LI-COR) antibodies. The protein bands were visualized by Odyssey Clx (LI-COR) and quantified with Image Studio Lite.

##### Small RNA duplex loading assay

For the small RNA duplex loading assays using recombinant proteins, 0.5 μM small RNA duplex with 5’ ^32^P-labeled guide was incubated for 1 hour, at either 25 or 37°C, with 7.5 μM recombinant FLAG-AGO2 or mutant in 1x trimming buffer supplemented with 0.1 mg/mL BSA. Pre-equilibrated anti-FLAG^®^ M2 Magnetic Beads were added into the mixture at the same temperature and incubated for 1 hour to allow binding of protein-RNA complex to the beads. The beads were then washed 8 times with 10 beads volume of wash buffer (50 mM Tris-HCl pH 7.5, 300 mM NaCl, 0.05% NP-40) using a magnetic stand. Each sample was then split into 2 halves, with one half quenched with 2x quenching buffer followed by incubation at 90°C for 3 min and running on a 20% denaturing gel. The other half was used for RNA extraction using phenol chloroform and ethanol precipitation. Extracted RNAs were annealed the same way as described above and then resolved on a 16% native polyacrylamide gel. Phosphor images were taken by a Typhoon Imager (GE Healthcare) and band intensity was quantified using Image Lab (Bio-Rad). The results were graphed using GraphPad Prism (GraphPad Software, Inc.).

For the small RNA duplex loading assays using immunopurified proteins, 40-60 mg of HEK293T cell pellet from transfection of pCAGEN-FLAG-AGO2-WT and −Δα14 was resuspended with 2x volume of 1x hypotonic buffer (20 mM HEPES-KOH, pH 7.4, 10 mM NaCl, 1.5 mM MgCl_2_ and 0.1% (v/v) Tween-20, 5 mM DTT, 1× EDTA-free complete protease inhibitor tablets), briefly stirred with a pipette, and incubated on ice for 20 min. The samples were then centrifuged at 21,130 ×g for 20 min at 4°C. The supernatant was transferred to a new 1.5 mL tube. 10 μL of M2 magnetic beads were washed 3 times with 100 μL of 1x PBS, then incubated with 10 μL of cell lysate from FLAG-AGO2-WT or −Δα14. The samples were incubated at room temperature for 1 hour while rotating. The beads were washed 8 times with 100 μL of wash buffer, followed by 3 washes with 200 μL of 1x PBS and 1 wash with 100 μL of 1x trimming buffer. 10 μL of 1x trimming buffer was added into each sample. 2 μL of 50 nM spiked siRNA duplex was then added into each aliquot for both FLAG-AGO2-WT and −Δα14, followed by incubation on an Eppendorf ThermoMixer at 25°C or 37°C with shaking at 1,400 rpm for 1 hour. After 8 washes, each sample was split into 2 halves: one half for the denaturing gel and the other for RNA extraction, followed by a native gel. 15 μL of 2x quenching buffer was added to one half of each sample, and the mixture was incubated for 3 min at 90°C. A 20% denaturing gel was used to resolve the RNA. 10% of each RNA added to the reactions was loaded as input. The gel was dried using a gel dryer. After cooling, the gel was exposed to the phosphor imager screen for overnight. Phosphor images were taken by a Typhoon Imager (GE Healthcare) and band intensity was quantified using Image Lab (Bio-Rad). The other half of each sample was subjected to phenol-chloroform extraction and ethanol precipitation, followed by re-annealing in 1x trimming buffer and 16% polyacrylamide native gel run in 0.5x TBE. Perform the same procedures for the comparison of miR-20a duplex loading and RISC maturation of FLAG-AGO2-WT, N mutant, and −Δα7 at 37°C.

##### 3’ end ^32^P labeling of passenger RNA

First, cytidine-3’-monophosphate was radiolabeled using *γ*-^32^P ATP (3,000 Ci mmol^−1^; Revvity) with T4 Polynucleotide Kinase (Thermo Scientific^™^) at 37°C for 1.5 hours, followed by inactivation at 65°C for 30 min and 90°C for 5 min. Second, the resultant ^32^P-pCp in the reaction was then used for 3’ end labeling of passenger RNA using T4 RNA ligase (NEB). Unincorporated ^32^P-pCp and *γ*-^32^P ATP were removed using MicroSpin^™^ G-25 columns (Cytiva).

##### Passenger ejection assay in the absence of Mg^2+^

miR-20a or let-7a duplex with passenger strand 3’ end ^32^P-labeled was loaded onto purified FLAG-AGO2 at 37°C as described above. Each reaction contained 5 μL of magnetic beads, 2.5 pmol of RNA duplex, and 37.5 pmol of recombinant FLAG-AGO2. After loading at 37°C, all samples were washed 8 times with 100 μL of wash buffer, 4 times with 200 μL of 1x PBS, and equilibrated once with 10 μL of 1x trimming buffer. 10 μL of 1x trimming buffer was added to each sample, which was then placed on ice. No target or 40 pmol of target was added to corresponding reaction (Figures 6A–6C). At 0, 30, and 50 min (corresponding to 60, 30, and 10 min incubation), the corresponding samples were moved from ice to a 37°C ThermoMixer (Eppendorf) at 1,400 rpm for passenger ejection. One sample was kept on ice and treated as the 0 min sample for the 37°C incubation. At 60 min, all samples were moved back to ice. For the passenger ejection by the F2L3 mutant, the assays were carried out the same way with incubation at 37°C for 0 or 30 min with WT AGO2 as control performed in parallel (Figure 6D). A magnetic stand was used to help transfer the supernatant to new tubes. The magnetic beads were then washed 8 times with 100 μL of wash buffer. 10 μL and 15 μL of 2x quenching buffer were added to the supernatant and beads, respectively. Passenger ejection assays for AGO1, 3, and 4 were performed the same way. miR-20a duplex (guide:passenger = 1:0.9) with the same 3’ end labeling of passenger incubated with 40x miR-20a target in the absence of AGO2, followed by a native gel, was used as one negative control. Another control experiment using miR-20a duplex with the guide strand 5’ ^32^P-labeled was performed similarly with incubation at 37°C for 0 or 90 min after addition of a target RNA. For denaturing gels, the samples were heated at 90°C for 3 min before loading. 20% glycerol was added to each native gel sample before loading. All gels were dried on a gel dryer. Phosphor images were taken by a Typhoon Imager (GE Healthcare), and band intensity was quantified using Image Lab (Bio-Rad). The results were graphed using GraphPad Prism (GraphPad Software, Inc.).

##### Passenger ejection assay in the presence of Mg^2+^

miR-20a duplex (guide:passenger = 1:0.9) with 3’ ^32^P-labeled non-modified or modified passenger was loaded onto FLAG-AGO2 WT as described above. After loading and IP, all samples were kept on ice and washed 8 times with 100 μL of wash buffer, 4 times with 200 μL of 1x PBS, and equilibrated once with 10 μL of 1x trimming buffer supplemented with 5 mM MgCl_2_. 10 μL of cold 1x trimming buffer supplemented with 5 mM MgCl_2_ was added to the corresponding sample. 40 pmol of target was added to the corresponding reactions. For controls, no target was added. The corresponding samples were moved from ice to a 37°C ThermoMixer (Eppendorf) at 1,400 rpm for passenger cleavage and ejection. For 0 min 37°C incubation samples, the reactions were kept on ice. At 30 min, all samples were moved back on ice. A magnetic stand was used to help transfer the supernatant to new tubes. The magnetic beads were then washed 8 times on ice with 100 μL of wash buffer. 10 μL and 15 μL of 2x quenching buffer were added to the supernatant and the beads, respectively. All samples were heated at 90°C for 3 min before loading onto 20% denaturing gels. All gels were dried on a gel dryer. Phosphor images were taken by a Typhoon Imager (GE Healthcare), and band intensity was quantified using Image Lab (Bio-Rad). The results were graphed using GraphPad Prism (GraphPad Software, Inc.).

### QUANTIFICATION AND STATISTICAL ANALYSIS

Statistical analyses were performed using GraphPad Prism (version 10.6.1). Unpaired t-tests (two-tailed) were used to analyze data from two independent groups, while a one-way ANOVA test (Dunnett’s post-hoc) was used to analyze the means of data from three or more groups. All quantitative data was performed in triplicate and depicted as mean ± SD, with P-values represented as follows: * (P < 0.05); ** (P < 0.01); *** (P < 0.001); **** (P < 0.0001); ns, not significant.

## Supplementary Material

MMC1

MMC6

MMC7

MMC2

MMC3

MMC5

MMC4

MMC8

Supplemental information can be found online at https://doi.org/10.1016/j.molcel.2026.04.029.

## Figures and Tables

**Table 1.** Cryo-EM data collection, refinement, and validation statistics

	#1 pri-RISC (EMDB-73099) (PDB: 9YM9)	#2 vise-RISC (EMDB-73100) (PDB: 9YMA)	#3 wedg-RISC (EMDB-73101) (PDB: 9YMB)	#4 ej-RISC (EMDB-73102) (PDB: 9YMC)	#5 mat-RISC (EMDB-73103) (PDB: 9YMD)	#6 target-bound mat-RISC (EMDB-75039) (PDB: 10BE)
Data collection and processing						
Magnification	81,000	81,000	81,000	105,000	105,000	105,000
Voltage (kV)	300	300	300	300	300	300
Electron exposure (e^−^/Å^2^)	50	50	50	50	50	50
Defocus range (μm)	−0.6 to −1.6	−0.6 to −1.6	−0.6 to −1.6	−0.8 to −2.4	−0.8 to −2.4	−0.8 to −2.2
Pixel size (Å)	0.426	0.426	0.426	0.835	0.835	0.7296
Symmetry imposed	C1	C1	C1	C1	C1	C1
Initial particle images (no.)	2,702,925	2,702,925	2,702,925	3,303,317	3,303,317	2,752,920
Final particle images (no.)	203,008	64,361	64,344	66,044	63,912	200,460
Map resolution (Å)	3.79	3.87	3.77	3.28	3.45	3.02
FSC threshold	0.143	0.143	0.143	0.143	0.143	0.143
Map resolution range (Å)	3.17–6.79	3.20–6.31	3.29–5.57	2.79–6.85	2.73–7.66	2.68–3.80
Refinement						
Initial model used (PDB code)	AlphaFold3	AlphaFold3	AlphaFold3	AlphaFold3	AlphaFold3, 4W5N	4W5N, 4W5O
Model resolution (Å)	3.79	3.87	3.77	3.28	3.45	3.02
FSC threshold	0.143	0.143	0.143	0.143	0.143	0.143
Model resolution range (Å)	3.17–6.79	3.20–6.31	3.29–5.57	2.79–6.85	2.73–7.66	2.68–3.80
Map sharpening *B* factor (Å^2^)	156.3	126.92	131.56	104.41	108.99	134.4
Model composition						
Non-hydrogen atoms	4,481	6,062	5,628	6,561	6,299	6757
Protein residues	455	645	656	768	758	792
RNA	41	42	18	20	11	19
*B* factors (Å^2^)						
Protein	92.88	145.63	148.06	115.57	60.24	73.01
RNA	52.09	191.45	135.36	124.39	120.51	59.71
RMS deviations						
Bond lengths (Å)	0.003	0.003	0.003	0.003	0.003	0.002
Bond angles (°)	0.677	0.545	0.648	0.683	0.524	0.463
Validation						
MolProbity score	2.48	2.40	2.29	2.37	1.93	1.47
Clashscore	14.14	14.09	10.46	9.64	7.97	6.43
Poor rotamers (%)	3.31	3.87	3.65	3.55	1.80	0.00
Ramachandran plot						
Favored (%)	93.54	95.75	95.36	93.14	95.70	97.42
Allowed (%)	6.46	4.09	4.64	6.86	4.30	2.58
Disallowed (%)	0.00	0.16	0.00	0.00	0.00	0.00

**Table:** KEY RESOURCES TABLE

REAGENT or RESOURCE	SOURCE	IDENTIFIER
Antibodies		
Anti-FLAG^®^ M2 Magnetic Beads	Millipore Sigma	Cat# M8823; RRID: AB_2637089
Bacterial and virus strains		
DH5α MAX Efficiency competent cells	Invitrogen^™^	Cat # 18258012
DH10bac MAX Efficiency Invitrogen	Invitrogen^™^	Cat # 1492020
Rosetta 2(DE3)pLysS	Millipore Sigma	Cat # 71403-3
Chemicals, peptides, and recombinant proteins		
AGO2	This paper	N/A
FLAG-AGO2	This paper	N/A
FLAG-AGO2-F2L3 (F294A and L339A)	This paper	N/A
FLAG-AGO2-L192P	This paper	N/A
FLAG-AGO2-ΔF182	This paper	N/A
FLAG-AGO1	This paper	N/A
FLAG-AGO3	This paper	N/A
FLAG-AGO4	This paper	N/A
SUMO-ISG20	This paper	N/A
Pierce^™^ Monomeric Avidin Agarose	Thermo Scientific^™^	Cat# 20228
SigmaFAST Protease Inhibitor Cocktail, EDTA-free	Millipore Sigma	Cat# S8830
Micrococcal nuclease	Takara	Cat# 2910A
UltraPure^™^ BSA (50 mg/mL)	Invitrogen^™^	Cat# AM2616
AccuGel 29:1 (40%)	National Diagnostics	Cat# EC-852
*γ*-^32^P ATP (3,000 Ci mmol-1)	Revvity	Cat# BLU002A250UC
T4 Polynucleotide Kinase (10 U/μL)	Thermo Scientific^™^	Cat# EK0031
RiboLock RNase Inhibitor (40 U/μL)	Thermo Scientific^™^	Cat# EO0381
T4 RNA Ligase 1 (ssRNA Ligase)	NEB	Cat# M0204S
FastAP Thermosensitive Alkaline Phosphatase	Thermo Scientific^™^	Cat# EF0651
Cytidine 3’-monophosphate	Cayman chemical	Cat# 33833
TransIT-X2 Dynamic Delivery System	Mirus Bio	Cat# MIR 6006
RIPA Buffer (10X)	Cell Signaling Technology	Cat# 9806
Critical commercial assays		
Bac-to-Bac^™^ Baculovirus Expression System	Gibco^™^	Cat# 10359016
Deposited data		
Human AGO2 pri-RISC	This paper	PDB: 9YM9
Human AGO2 vise-RISC	This paper	PDB: 9YMA
Human AGO2 wedg-RISC	This paper	PDB: 9YMB
Human AGO2 ej-RISC	This paper	PDB: 9YMC
Human AGO2 mat-RISC	This paper	PDB: 9YMD
Human AGO2 RISC with an 18-nt target	This paper	PDB: 10BE
Crystal structure of Human AGO2 with guide	Schirle et al.^45^	PDB: 4OLA
Crystal structure of Human AGO2-miR-20a complex	Elkayam et al.^60^	PDB: 4F3T
Crystal structure of Human AGO2 bound to a defined guide RNA	Schirle et al.^61^	PDB: 4W5N
Crystal structure of Human AGO2 bound to a guide and target RNA containing seed pairing from 2–9	Schirle et al.^61^	PDB: 4W5O
Crystal structure of Human AGO2 bound to a guide and target RNA containing seed pairing from 2–8	Schirle et al.^61^	PDB: 4W5Q
Crystal structure of Human AGO2 bound to a guide and target RNA containing seed pairing from 2–8	Schirle et al.^61^	PDB: 4W5R
Crystal structure of Human AGO2 bound to a guide and target RNA containing seed pairing from 2–7	Schirle et al.^61^	PDB: 4W5T
Crystal structure of Human AGO2 bound to miR-122 with seed and supplementary paired target	Sheu-Gruttadauria et al.^62^	PDB: 6N4O
Crystal structure of Human AGO2 bound to miR-27a and HSUR1 target RNA	Sheu-Gruttadauria et al.^63^	PDB: 6MFN
Crystal structure of Human AGO2 bound to miR-122 and a target RNA with two central mismatches	Sheu-Gruttadauria et al.^63^	PDB: 6MDZ
Crystal structure of Human AGO2 bound to miR-122 and a target RNA with three central mismatches	Sheu-Gruttadauria et al.^63^	PDB: 6MFR
Crystal structure of Human AGO2 bound to miR-122 and a target RNA with four central mismatches	Sheu-Gruttadauria et al.^63^	PDB: 6NIT
Cryo-EM structure of human AGO2 with guide and target complex in a fully paired, slicing-competent conformation	Mohamed et al.^65^	PDB: 9CMP
Cryo-EM structure of human AGO2 with a guide and a 12-nt target	Li et al.^66^	PDB: 9K6P
Cryo-EM structure of human AGO2 with a guide and a 14-nt target	Li et al.^66^	PDB: 9K6R
Cryo-EM structure of human AGO2 with a guide and a 19-nt target	Li et al.^66^	PDB: 9K6S
Cryo-EM structure of human AGO2 with a guide and a 21-nt target	Li et al.^66^	PDB: 9K6T
Raw images of gels and blots	This paper	Mendeley Data: 10.17632/hr73mdy8gw.1
Experimental models: Cell lines		
HEK293T	ATCC	Cat# CRL-1573
sf9	Expression Systems	Cat# 94-001F
T.ni	Expression Systems	Cat# 94-002F
Oligonucleotides		
See Table S1	This paper	N/A
Recombinant DNA		
pFastBac^™^HTB-AGO2	Park et al.^72^	N/A
pFastBac^™^HTB-FLAG-AGO2	Park et al.^72^	N/A
pFastBac^™^HTB-FLAG-AGO2-F2L3 (F294A and L339A)	This paper	N/A
pFastBac^™^HTB-FLAG-AGO1	Nakanishi et al.^78^	N/A
pFastBac^™^HTB-FLAG-AGO3	Park et al.^72^	N/A
pFastBac^™^HTB-FLAG-AGO4	Park et al.^46^	N/A
pFastBac^™^HTB-FLAG-AGO2-L192P	This paper	N/A
pFastBac^™^HTB-FLAG-AGO2-ΔF182	This paper	N/A
pRSF-SUMO-ISG20	Sim, et al.^42^	N/A
pCAGEN-FLAG-AGO2	Park et al.^72^	N/A
pCAGEN-FLAG-AGO2-Δα7 (L356-T368 to 5 glycines)	This paper	N/A
pCAGEN-FLAG-AGO2-Δα14 (T556-N562 deletion)	This paper	N/A
pCAGEN-FLAG-AGO2 N mutant (K62A, K65A, R68A, R69A)	This paper	N/A
Software and algorithms		
Image Lab (version 6.0.1 build 7)	BioRad	https://www.bio-rad.com/en-us/product/image-lab-software?ID=KRE6P5E8Z
Prism (Version 10.6.1 (799))	GraphPad	https://www.graphpad.com/features
CryoSPARC V.4	Punjani et al.^80^	https://cryosparc.com
Phenix (Version 1.21-5207-000)	Adams et al.^81^	https://phenix-online.org/
Coot (0.9.8.93 EL)	Emsley et al.^82^	https://www2.mrc-lmb.cam.ac.uk/personal/pemsley/coot/
ChimeraX (Version 1.8)	Meng et al.^83^	https://www.cgl.ucsf.edu/chimerax/
Image Studio Lite (Version 5.2.5)	Li-COR	https://www.licor.com/bio/image-studio/
PyMOL (Version 2.5.4)	Schrö dinger, Inc.	https://pymol.org/2/

## Data Availability

The dataset and analytic code that support the findings of this study are available at: https://osf.io/tqycs/
Atomic coordinates for pri-, vise-, wedg-, ej-, mat-RISC, and mat-RISC-target have been deposited in the Protein data Bank (PDB: 9YM9, 9YMA, 9YMB, 9YMC, 9YMD, and 10BE), and cryo-EM density maps have been deposited in the Electron Microscopy Data Bank (EMD: EMD-73099, EMD-73100, EMD-73101, EMD-73102, EMD-73103, and EMD-75039). Uncropped images of gels and blots have been deposited into Mendeley data: 10.17632/hr73mdy8gw.1. These data are publicly available as of the date of publication. This paper does not report any original code. Any additional information required to reanalyze the data reported in this paper is available from the lead contact upon request.

## Highlights

- RISC assembly advances through discrete, drastic conformational changes
- AGO2 adopts an open conformation to load duplexes via the MID-PIWI lobe
- α14 directs end selection by sensing duplex thermodynamic asymmetry
- Target binding drives passenger ejection even after passenger cleavage

## References

[1] Shabalina SA, and Koonin EV (2008). Origins and evolution of eukaryotic RNA interference. Trends Ecol. Evol 23, 578–587. 10.1016/j.tree.2008.06.005. 18715673 PMC2695246

[2] Iwakawa HO, and Tomari Y (2022). Life of RISC: Formation, action, and degradation of RNA-induced silencing complex. Mol. Cell 82, 30–43. 10.1016/j.molcel.2021.11.026. 34942118

[3] Bartel DP (2018). Metazoan MicroRNAs. Cell 173, 20–51. 10.1016/j.cell.2018.03.006. 29570994 PMC6091663

[4] Shang R, Lee S, Senavirathne G, and Lai EC (2023). microRNAs in action: biogenesis, function and regulation. Nat. Rev. Genet 24, 816–833. 10.1038/s41576-023-00611-y. 37380761 PMC11087887

[5] Nakanishi K (2022). Anatomy of four human Argonaute proteins. Nucleic Acids Res. 50, 6618–6638. 10.1093/nar/gkac519. 35736234 PMC9262622

[6] Nakanishi K (2016). Anatomy of RISC: how do small RNAs and chaperones activate Argonaute proteins? WIREs RNA 7, 637–660. 10.1002/wrna.1356. 27184117 PMC5084781

[7] Yamaguchi S, Naganuma M, Nishizawa T, Kusakizako T, Tomari Y, Nishimasu H, and Nureki O (2022). Structure of the Dicer-2-R2D2 heterodimer bound to a small RNA duplex. Nature 607, 393–398. 10.1038/s41586-022-04790-2. 35768503 PMC9279153

[8] Tomari Y, Matranga C, Haley B, Martinez N, and Zamore PD (2004). A protein sensor for siRNA asymmetry. Science 306, 1377–1380. 10.1126/science.1102755. 15550672

[9] MacRae IJ, Ma E, Zhou M, Robinson CV, and Doudna JA (2008). In vitro reconstitution of the human RISC-loading complex. Proc. Natl. Acad. Sci. USA 105, 512–517. 10.1073/pnas.0710869105. 18178619 PMC2206567

[10] Gregory RI, Chendrimada TP, Cooch N, and Shiekhattar R (2005). Human RISC couples microRNA biogenesis and posttranscriptional gene silencing. Cell 123, 631–640. 10.1016/j.cell.2005.10.022. 16271387

[11] Murchison EP, Partridge JF, Tam OH, Cheloufi S, and Hannon GJ (2005). Characterization of Dicer-deficient murine embryonic stem cells. Proc. Natl. Acad. Sci. USA 102, 12135–12140. 10.1073/pnas.0505479102. 16099834 PMC1185572

[12] Schwarz DS, Hutvágner G, Du T, Xu Z, Aronin N, and Zamore PD (2003). Asymmetry in the assembly of the RNAi enzyme complex. Cell 115, 199–208. 10.1016/s0092-8674(03)00759-1. 14567917

[13] Khvorova A, Reynolds A, and Jayasena SD (2003). Functional siRNAs and miRNAs exhibit strand bias. Cell 115, 209–216. 10.1016/s0092-8674(03)00801-8. 14567918

[14] Betancur JG, and Tomari Y (2012). Dicer is dispensable for asymmetric RISC loading in mammals. RNA 18, 24–30. 10.1261/rna.029785.111. 22106413 PMC3261740

[15] Iwasaki S, Kobayashi M, Yoda M, Sakaguchi Y, Katsuma S, Suzuki T, and Tomari Y (2010). Hsc70/Hsp90 chaperone machinery mediates ATP-dependent RISC loading of small RNA duplexes. Mol. Cell 39, 292–299. 10.1016/j.molcel.2010.05.015. 20605501

[16] Deerberg A, Willkomm S, and Restle T (2013). Minimal mechanistic model of siRNA-dependent target RNA slicing by recombinant human Argonaute 2 protein. Proc. Natl. Acad. Sci. USA 110, 17850–17855. 10.1073/pnas.1217838110. 24101500 PMC3816469

[17] Frank F, Sonenberg N, and Nagar B (2010). Structural basis for 5′-nucleotide base-specific recognition of guide RNA by human AGO2. Nature 465, 818–822. 10.1038/nature09039. 20505670

[18] Suzuki HI, Katsura A, Yasuda T, Ueno T, Mano H, Sugimoto K, and Miyazono K (2015). Small-RNA asymmetry is directly driven by mammalian Argonautes. Nat. Struct. Mol. Biol 22, 512–521. 10.1038/nsmb.3050. 26098316

[19] Park JH, and Shin C (2015). Slicer-independent mechanism drives small-RNA strand separation during human RISC assembly. Nucleic Acids Res. 43, 9418–9433. 10.1093/nar/gkv937. 26384428 PMC4627090

[20] Fire A, Xu S, Montgomery MK, Kostas SA, Driver SE, and Mello CC (1998). Potent and specific genetic interference by double-stranded RNA in Caenorhabditis elegans. Nature 391, 806–811. 10.1038/35888. 9486653

[21] Elbashir SM, Harborth J, Lendeckel W, Yalcin A, Weber K, and Tuschl T (2001). Duplexes of 21-nucleotide RNAs mediate RNA interference in cultured mammalian cells. Nature 411, 494–498. 10.1038/35078107. 11373684

[22] Hammond SM, Bernstein E, Beach D, and Hannon GJ (2000). An RNA-directed nuclease mediates post-transcriptional gene silencing in Drosophila cells. Nature 404, 293–296. 10.1038/35005107. 10749213

[23] Lee RC, and Ambros V (2001). An extensive class of small RNAs in Caenorhabditis elegans. Science 294, 862–864. 10.1126/science.1065329. 11679672

[24] Lai EC (2002). Micro RNAs are complementary to 3′ UTR sequence motifs that mediate negative post-transcriptional regulation. Nat. Genet 30, 363–364. 10.1038/ng865. 11896390

[25] Bartel DP (2004). MicroRNAs. Cell 116, 281–297. 10.1016/s0092-8674(04)00045-5. 14744438

[26] Doench JG, and Sharp PA (2004). Specificity of microRNA target selection in translational repression. Genes Dev. 18, 504–511. 10.1101/gad.1184404. 15014042 PMC374233

[27] Brennecke J, Stark A, Russell RB, and Cohen SM (2005). Principles of microRNA-target recognition. PLOS Biol. 3, e85. 10.1371/journal.pbio.0030085. 15723116 PMC1043860

[28] Lim LP, Lau NC, Garrett-Engele P, Grimson A, Schelter JM, Castle J, Bartel DP, Linsley PS, and Johnson JM (2005). Microarray analysis shows that some microRNAs downregulate large numbers of target mRNAs. Nature 433, 769–773. 10.1038/nature03315. 15685193

[29] Bitetti A, Mallory AC, Golini E, Carrieri C, Carreño Gutiérrez H, Perlas E, Pérez-Rico YA, Tocchini-Valentini GP, Enright AJ, Norton WHJ, (2018). MicroRNA degradation by a conserved target RNA regulates animal behavior. Nat. Struct. Mol. Biol 25, 244–251. 10.1038/s41594-018-0032-x. 29483647

[30] Ghini F, Rubolino C, Climent M, Simeone I, Marzi MJ, and Nicassio F (2018). Endogenous transcripts control miRNA levels and activity in mammalian cells by target-directed miRNA degradation. Nat. Commun 9, 3119. 10.1038/s41467-018-05182-9. 30087332 PMC6081425

[31] Kleaveland B, Shi CY, Stefano J, and Bartel DP (2018). A Network of Noncoding Regulatory RNAs Acts in the Mammalian Brain. Cell 174, 350–362.e17. 10.1016/j.cell.2018.05.022. 29887379 PMC6559361

[32] Ameres SL, Horwich MD, Hung JH, Xu J, Ghildiyal M, Weng Z, and Zamore PD (2010). Target RNA-directed trimming and tailing of small silencing RNAs. Science 328, 1534–1539. 10.1126/science.1187058. 20558712 PMC2902985

[33] Cazalla D, Yario T, and Steitz JA (2010). Down-regulation of a host microRNA by a Herpesvirus saimiri noncoding RNA. Science 328, 1563–1566. 10.1126/science.1187197. 20558719 PMC3075239

[34] Shi CY, Kingston ER, Kleaveland B, Lin DH, Stubna MW, and Bartel DP (2020). The ZSWIM8 ubiquitin ligase mediates target-directed microRNA degradation. Science 370. 10.1126/science.abc9359. PMC8356967 33184237

[35] Han J, LaVigne CA, Jones BT, Zhang H, Gillett F, and Mendell JT (2020). A ubiquitin ligase mediates target-directed microRNA decay independently of tailing and trimming. Science 370. 10.1126/science.abc9546. PMC8177725 33184234

[36] Schwanhäusser B, Busse D, Li N, Dittmar G, Schuchhardt J, Wolf J, Chen W, and Selbach M (2011). Global quantification of mammalian gene expression control. Nature 473, 337–342. 10.1038/nature10098. 21593866

[37] Marinov GK, Williams BA, McCue K, Schroth GP, Gertz J, Myers RM, and Wold BJ (2014). From single-cell to cell-pool transcriptomes: stochasticity in gene expression and RNA splicing. Genome Res. 24, 496–510. 10.1101/gr.161034.113. 24299736 PMC3941114

[38] Chatterjee S, Fasler M, Büssing I, and Großhans H (2011). Target-mediated protection of endogenous microRNAs in C. elegans. Dev. Cell 20, 388–396. 10.1016/j.devcel.2011.02.008. 21397849

[39] Pitchiaya S, Heinicke LA, Park JI, Cameron EL, and Walter NG (2017). Resolving Subcellular miRNA Trafficking and Turnover at Single-Molecule Resolution. Cell Rep. 19, 630–642. 10.1016/j.celrep.2017.03.075. 28423324 PMC5482240

[40] Rand TA, Petersen S, Du F, and Wang X (2005). Argonaute2 cleaves the anti-guide strand of siRNA during RISC activation. Cell 123, 621–629. 10.1016/j.cell.2005.10.020. 16271385

[41] Matranga C, Tomari Y, Shin C, Bartel DP, and Zamore PD (2005). Passenger-strand cleavage facilitates assembly of siRNA into Ago2-containing RNAi enzyme complexes. Cell 123, 607–620. 10.1016/j.cell.2005.08.044. 16271386

[42] Sim G, Kehling AC, Park MS, Secor J, Divoky C, Zhang H, Malhotra N, Bhagdikar D, Abd El-Wahab EW, and Nakanishi K (2022). Manganese-dependent microRNA trimming by 3′→5′ exonucleases generates 14-nucleotide or shorter tiny RNAs. Proc. Natl. Acad. Sci. USA 119, e2214335119. 10.1073/pnas.2214335119. 36508664 PMC9907110

[43] Sim G, Kehling AC, Park MS, Divoky C, Zhang H, Malhotra N, Secor J, and Nakanishi K (2023). Determining the defining lengths between mature microRNAs/small interfering RNAs and tinyRNAs. Sci. Rep 13, 19761. 10.1038/s41598-023-46562-6. 37957252 PMC10643408

[44] Li Z, Xu Q, Zhong J, Zhang Y, Zhang T, Ying X, Lu X, Li X, Wan L, Xue J, (2025). Structural insights into RNA cleavage by PIWI Argonaute. Nature 639, 250–259. 10.1038/s41586-024-08438-1. 39814893

[45] Schirle NT, and MacRae IJ (2012). The crystal structure of human Argonaute2. Science 336, 1037–1040. 10.1126/science.1221551. 22539551 PMC3521581

[46] Park MS, Araya-Secchi R, Brackbill JA, Phan HD, Kehling AC, Abd El-Wahab EW, Dayeh DM, Sotomayor M, and Nakanishi K (2019). Multidomain Convergence of Argonaute during RISC Assembly Correlates with the Formation of Internal Water Clusters. Mol. Cell 75, 725–740.e6. 10.1016/j.molcel.2019.06.011. 31324450 PMC6707842

[47] Sano M, Sierant M, Miyagishi M, Nakanishi M, Takagi Y, and Sutou S (2008). Effect of asymmetric terminal structures of short RNA duplexes on the RNA interference activity and strand selection. Nucleic Acids Res. 36, 5812–5821. 10.1093/nar/gkn584. 18782830 PMC2566866

[48] Ma JB, Ye K, and Patel DJ (2004). Structural basis for overhang-specific small interfering RNA recognition by the PAZ domain. Nature 429, 318–322. 10.1038/nature02519. 15152257 PMC4700412

[49] Schwarz DS, Hutvágner G, Haley B, and Zamore PD (2002). Evidence that siRNAs function as guides, not primers, in the Drosophila and human RNAi pathways. Mol. Cell 10, 537–548. 10.1016/s1097-2765(02)00651-2. 12408822

[50] Kwak PB, and Tomari Y (2012). The N domain of Argonaute drives duplex unwinding during RISC assembly. Nat. Struct. Mol. Biol 19, 145–151. 10.1038/nsmb.2232. 22233755

[51] Yoda M, Kawamata T, Paroo Z, Ye X, Iwasaki S, Liu Q, and Tomari Y (2010). ATP-dependent human RISC assembly pathways. Nat. Struct. Mol. Biol 17, 17–23. 10.1038/nsmb.1733. 19966796 PMC2915567

[52] Klum SM, Chandradoss SD, Schirle NT, Joo C, and MacRae IJ (2018). Helix-7 in Argonaute2 shapes the microRNA seed region for rapid target recognition. EMBO J. 37, 75–88. 10.15252/embj.201796474. 28939659 PMC5753032

[53] Yang A, Shao TJ, Bofill-De Ros X, Lian C, Villanueva P, Dai L, and Gu S (2020). AGO-bound mature miRNAs are oligouridylated by TUTs and subsequently degraded by DIS3L2. Nat. Commun 11, 2765. 10.1038/s41467-020-16533-w. 32488030 PMC7265490

[54] Schalk A, Cousin MA, Dsouza NR, Challman TD, Wain KE, Powis Z, Minks K, Trimouille A, Lasseaux E, Lacombe D, (2021). De novo coding variants in the AGO1 gene cause a neurodevelopmental disorder with intellectual disability. J. Med. Genet 59, 965–975. 10.1136/jmedgenet-2021-107751. 34930816 PMC9241146

[55] Lessel D, Zeitler DM, Reijnders MRF, Kazantsev A, Hassani Nia F, Bartholomäus A, Martens V, Bruckmann A, Graus V, McConkie-Rosell A, (2020). Germline AGO2 mutations impair RNA interference and human neurological development. Nat. Commun 11, 5797. 10.1038/s41467-020-19572-5. 33199684 PMC7670403

[56] Duan Y, Li L, Panzade GP, Piton A, Zinovyeva A, and Ambros V (2024). Modeling neurodevelopmental disorder-associated human AGO1 mutations in Caenorhabditis elegans Argonaute alg-1. Proc. Natl. Acad. Sci. USA 121, e2308255121. 10.1073/pnas.2308255121. 38412125 PMC10927592

[57] Savidge A, Zhang H, Annasaheb Adhav V, Kehling AC, Sim G, Shen Z, Fu TM, and Nakanishi K (2025). Neurodevelopmental disorder-linked Argonaute mutations permit delayed RISC formation and unusual shortening of miRNAs by 3′→5′ trimming. Proc. Natl. Acad. Sci. USA 122, e2524644122. 10.1073/pnas.2524644122. 41237208 PMC12646310

[58] Liu N, and Wang HW (2023). Better Cryo-EM Specimen Preparation: How to Deal with the Air-Water Interface? J. Mol. Biol 435, 167926. 10.1016/j.jmb.2022.167926. 36563741

[59] Hirst IJ, Thomas WJR, Davies RA, and Muench SP (2024). CryoEM grid preparation: a closer look at advancements and impact of preparation mode and new approaches. Biochem. Soc. Trans 52, 1529–1537. 10.1042/BST20231553. 38864435 PMC11346429

[60] Elkayam E, Kuhn CD, Tocilj A, Haase AD, Greene EM, Hannon GJ, and Joshua-Tor L (2012). The structure of human argonaute-2 in complex with miR-20a. Cell 150, 100–110. 10.1016/j.cell.2012.05.017. 22682761 PMC3464090

[61] Schirle NT, Sheu-Gruttadauria J, and MacRae IJ (2014). Structural basis for microRNA targeting. Science 346, 608–613. 10.1126/science.1258040. 25359968 PMC4313529

[62] Sheu-Gruttadauria J, Xiao Y, Gebert LF, and MacRae IJ (2019). Beyond the seed: structural basis for supplementary microRNA targeting by human Argonaute2. EMBO J. 38, e101153. 10.15252/embj.2018101153. 31268608 PMC6600645

[63] Sheu-Gruttadauria J, Pawlica P, Klum SM, Wang S, Yario TA, Schirle Oakdale NT, Steitz JA, and MacRae IJ (2019). Structural Basis for Target-Directed MicroRNA Degradation. Mol. Cell 75, 1243–1255.e7. 10.1016/j.molcel.2019.06.019. 31353209 PMC6754277

[64] Sarkar S, Gebert LFR, and MacRae IJ (2024). Structural basis for gene silencing by siRNAs in humans. bioRxiv. 10.1101/2024.12.05.627081.

[65] Mohamed AA, Wang PY, Bartel DP, and Vos SM (2025). The structural basis for RNA slicing by human Argonaute2. Cell Rep. 44, 115166. 10.1016/j.celrep.2024.115166. 39932188 PMC11893014

[66] Li Z, Xu Q, Zhang Y, Zhong J, Zhang T, Xue J, Liu S, Gao H, Zhang ZZZ, Wu J, (2025). Mechanistic insights into RNA cleavage by human Argonaute2-siRNA complex. Cell Res. 35, 453–464. 10.1038/s41422-025-01114-7. 40240484 PMC12134215

[67] Miyoshi K, Tsukumo H, Nagami T, Siomi H, and Siomi MC (2005). Slicer function of Drosophila Argonautes and its involvement in RISC formation. Genes Dev. 19, 2837–2848. 10.1101/gad.1370605. 16287716 PMC1315391

[68] Leuschner PJF, Ameres SL, Kueng S, and Martinez J (2006). Cleavage of the siRNA passenger strand during RISC assembly in human cells. EMBO Rep. 7, 314–320. 10.1038/sj.embor.7400637. 16439995 PMC1456892

[69] Nakanishi K, Weinberg DE, Bartel DP, and Patel DJ (2012). Structure of yeast Argonaute with guide RNA. Nature 486, 368–374. 10.1038/nature11211. 22722195 PMC3853139

[70] Nakanishi K (2024). When Argonaute takes out the ribonuclease sword. J. Biol. Chem 300, 105499. 10.1016/j.jbc.2023.105499. 38029964 PMC10772731

[71] Elbashir SM, Martinez J, Patkaniowska A, Lendeckel W, and Tuschl T (2001). Functional anatomy of siRNAs for mediating efficient RNAi in Drosophila melanogaster embryo lysate. EMBO J. 20, 6877–6888. 10.1093/emboj/20.23.6877. 11726523 PMC125328

[72] Park MS, Phan HD, Busch F, Hinckley SH, Brackbill JA, Wysocki VH, and Nakanishi K (2017). Human Argonaute3 has slicer activity. Nucleic Acids Res. 45, 11867–11877. 10.1093/nar/gkx916. 29040713 PMC5714244

[73] Park MS, Sim G, Kehling AC, and Nakanishi K (2020). Human Argonaute2 and Argonaute3 are catalytically activated by different lengths of guide RNA. Proc. Natl. Acad. Sci. USA 117, 28576–28578. 10.1073/pnas.2015026117. 33122430 PMC7682322

[74] Zhang H, Sim G, Kehling AC, Adhav VA, Savidge A, Pastore B, Tang W, and Nakanishi K (2024). Target cleavage and gene silencing by Argonautes with cityRNAs. Cell Rep. 43, 114806. 10.1016/j.celrep.2024.114806. 39368090 PMC11533134

[75] Liu J, Carmell MA, Rivas FV, Marsden CG, Thomson JM, Song JJ, Hammond SM, Joshua-Tor L, and Hannon GJ (2004). Argonaute2 is the catalytic engine of mammalian RNAi. Science 305, 1437–1441. 10.1126/science.1102513. 15284456

[76] Meister G, Landthaler M, Patkaniowska A, Dorsett Y, Teng G, and Tuschl T (2004). Human Argonaute2 mediates RNA cleavage targeted by miRNAs and siRNAs. Mol. Cell 15, 185–197. 10.1016/j.molcel.2004.07.007. 15260970

[77] Hauptmann J, Dueck A, Harlander S, Pfaff J, Merkl R, and Meister G (2013). Turning catalytically inactive human Argonaute proteins into active slicer enzymes. Nat. Struct. Mol. Biol 20, 814–817. 10.1038/nsmb.2577. 23665583

[78] Nakanishi K, Ascano M, Gogakos T, Ishibe-Murakami S, Serganov AA, Briskin D, Morozov P, Tuschl T, and Patel DJ (2013). Eukaryote-specific insertion elements control human ARGONAUTE slicer activity. Cell Rep. 3, 1893–1900. 10.1016/j.celrep.2013.06.010. 23809764 PMC3757560

[79] Gu S, Jin L, Huang Y, Zhang F, and Kay MA (2012). Slicing-independent RISC activation requires the argonaute PAZ domain. Curr. Biol 22, 1536–1542. 10.1016/j.cub.2012.06.040. 22795694 PMC3604743

[80] Punjani A, Rubinstein JL, Fleet DJ, and Brubaker MA (2017). cryoSPARC: algorithms for rapid unsupervised cryo-EM structure determination. Nat. Methods 14, 290–296. 10.1038/nmeth.4169. 28165473

[81] Adams PD, Afonine PV, Bunkóczi G, Chen VB, Davis IW, Echols N, Headd JJ, Hung LW, Kapral GJ, Grosse-Kunstleve RW, (2010). PHENIX: a comprehensive Python-based system for macromolecular structure solution. Acta Crystallogr. D Biol. Crystallogr 66, 213–221. 10.1107/S0907444909052925. 20124702 PMC2815670

[82] Emsley P, Lohkamp B, Scott WG, and Cowtan K (2010). Features and development of Coot. Acta Crystallogr. D Biol. Crystallogr 66, 486–501. 10.1107/S0907444910007493. 20383002 PMC2852313

[83] Meng EC, Goddard TD, Pettersen EF, Couch GS, Pearson ZJ, Morris JH, and Ferrin TE (2023). UCSF ChimeraX: Tools for structure building and analysis. Protein Sci. 32, e4792. 10.1002/pro.4792. 37774136 PMC10588335

[84] Flores-Jasso CF, Salomon WE, and Zamore PD (2013). Rapid and specific purification of Argonaute-small RNA complexes from crude cell lysates. RNA 19, 271–279. 10.1261/rna.036921.112. 23249751 PMC3543083

