# Structural basis for RISC assembly of human Argonaute2

**DOI:** 10.1016/j.molcel.2026.07.003

**Published:** 2026-07-14

**Authors:** Huaqun Zhang, Vishal Annasaheb Adhav, Audrey C. Kehling, Andrew Savidge, Zhangfei Shen, Tian-Min Fu, Kotaro Nakanishi

Due to a typesetter error, all supplemental videos included in the originally published version of this article were inadvertently placed out of order, causing the videos to be misaligned with their respective titles. The order of the supplemental videos and their titles have now been corrected. The typesetters apologize for any inconvenience and confusion this may have caused.

