# Supplementary material for: Structural basis for RISC assembly of human Argonaute2
Source: Mol Cell. Author manuscript; Available in PMC 2026 Jun 26. (PMC13306894; doi:10.1016/j.molcel.2026.04.029)
Supplement: MMC1 [file NIHMS2174505-supplement-MMC1.pdf]

Molecular Cell, Volume *86*

Supplemental information

Structural basis for RISC assembly

# of human Argonaute2

Huaqun Zhang, Vishal Annasaheb Adhav, Audrey C. Kehling, Andrew Savidge, Zhangfei Shen, Tian-Min Fu, and Kotaro Nakanishi

Supplementary Information

Structural Basis for RISC Assembly of Human Argonaute2

Huaqun Zhang[1], Vishal Annasaheb Adhav[1], Audrey C. Kehling[1], Andrew Savidge[2,3], Zhangfei Shen[4], Tian-Min Fu[2,3,4,5,6], Kotaro Nakanishi[1,2,3,7,8] *

[1]Department of Chemistry and Biochemistry, The Ohio State University, Columbus, Ohio, 43210, USA.
[2]Ohio State Biochemistry Program, The Ohio State University, Columbus, Ohio, 43210, USA.
[3]Center for RNA Biology, The Ohio State University, Columbus, Ohio, 43210, USA.
[4]Department of Biological Chemistry and Pharmacology, The Ohio State University, Columbus, OH, 43210, USA.
[5]The Ohio State University Comprehensive Cancer Center, Columbus, OH, 43210, USA.
[6]Department of Pathology, UMass Chan Medical School, Worcester, MA, 01655, USA
[7]Molecular, Cellular and Developmental Biology, The Ohio State University, Columbus, Ohio, 43210, USA.
[8]Lead contact.
*Corresponding author. Email: nakanishi.9@osu.edu.

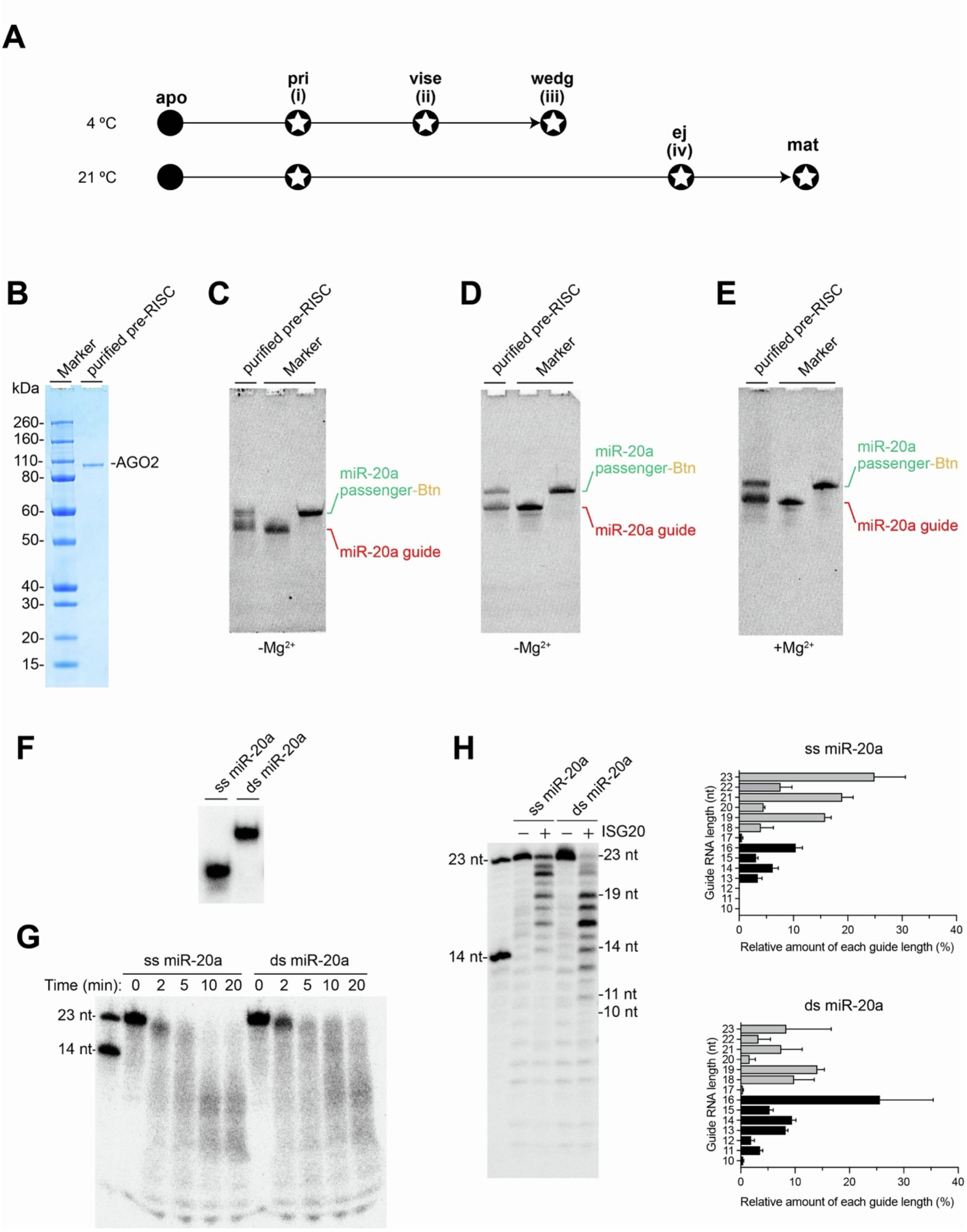

A
apo
pri (i)
vise (ii)
wedg (iii)
ej (iv)
mat
4 ºC
21 ºC
B
Marker
purified pre-RISC
kDa
260-
160-
110-
80-
60-
50-
40-
30-
20-
15-
-AGO2
C
purified pre-RISC
Marker
miR-20a passenger-Btn
miR-20a guide
-Mg2+
D
purified pre-RISC
Marker
miR-20a passenger-Btn
miR-20a guide
-Mg2+
E
purified pre-RISC
Marker
miR-20a passenger-Btn
miR-20a guide
+Mg2+
F
ss miR-20a
ds miR-20a
G
ss miR-20a
ds miR-20a
Time (min): 0 2 5 10 20 0 2 5 10 20
23 nt-
14 nt-
H
ss miR-20a
ds miR-20a
– + – + ISG20
23 nt-
14 nt-
-23 nt
-19 nt
-14 nt
-11 nt
-10 nt
ss miR-20a
ds miR-20a
Guide RNA length (nt)
Relative amount of each guide length (%)

Figure S1. AGO2 binds to a guide-passenger duplex to form a stable complex (related to Fig. 1)
(A) Simplified diagram of the different cryo-EM structures obtained from incubation of AGO2 and duplex at 4 and 21 ºC. States indicated with a star mark were reported in this study. The systematic names, State i-iv, are shown in parentheses.

(B) SDS-PAGE analysis of the purified duplex-bound AGO2.

(C-E) 20% denaturing gels, stained with SYBR Gold, of the RNA extracted from the purified duplex-bound AGO2 in (B) without treatment (C), after incubation on ice without Mg^{2+} for 3 days (D), or after incubation at room temperature with 5 mM Mg^{2+} for 1 hour (E).

(F) Single-stranded (ss) and double-stranded (ds) miR-20a used for in vitro guide trimming assay in (G) and (H). miR-20a guide strand was 5' ^{32}P-labeled.

(G) ISG20 trimmed both ss and ds RNAs. 20% denaturing gel of the ss or ds miR-20a after incubation with ISG20 for the indicated time.

(H) ISG20 trimming of ss and ds miR-20a loaded to FLAG-AGO2. (left) A representative gel image of the in vitro guide trimming assay. FLAG-AGO2 was incubated with 23-nt miR-20a (ss miR-20a) or an siRNA-like duplex of miR-20a (ds miR-20a), followed by trimming with ISG20. (right) Relative amounts of different lengths of the trimmed ss miR-20a (top) and ds miR-20a (bottom).

Assays in (F-H) were performed in triplicate, and data in (H) are depicted as the mean ± SD.

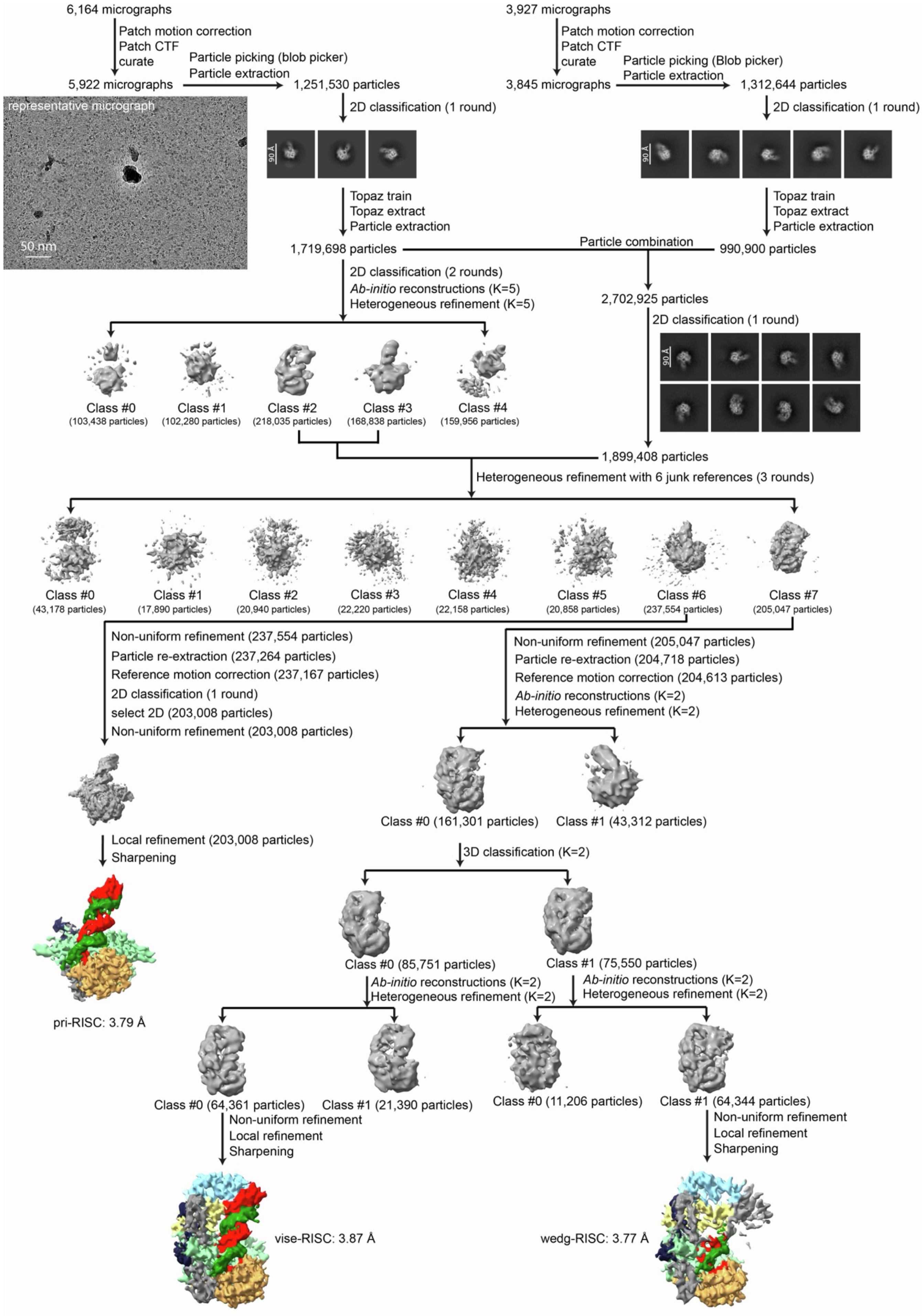

6,164 micrographs
Patch motion correction
Patch CTF
curate
5,922 micrographs
Particle picking (blob picker)
Particle extraction
1,251,530 particles
representative micrograph
50 nm
2D classification (1 round)
90 Å
Topaz train
Topaz extract
Particle extraction
1,719,698 particles
3,927 micrographs
Patch motion correction
Patch CTF
curate
3,845 micrographs
Particle picking (Blob picker)
Particle extraction
1,312,644 particles
2D classification (1 round)
Topaz train
Topaz extract
Particle extraction
990,900 particles
Particle combination
2,702,925 particles
2D classification (1 round)
2D classification (2 rounds)
Ab-initio reconstructions (K=5)
Heterogeneous refinement (K=5)
Class #0 (103,438 particles)
Class #1 (102,280 particles)
Class #2 (218,035 particles)
Class #3 (168,838 particles)
Class #4 (159,956 particles)
1,899,408 particles
Heterogeneous refinement with 6 junk references (3 rounds)
Class #0 (43,178 particles)
Class #1 (17,890 particles)
Class #2 (20,940 particles)
Class #3 (22,220 particles)
Class #4 (22,158 particles)
Class #5 (20,858 particles)
Class #6 (237,554 particles)
Class #7 (205,047 particles)
Non-uniform refinement (237,554 particles)
Particle re-extraction (237,264 particles)
Reference motion correction (237,167 particles)
2D classification (1 round)
select 2D (203,008 particles)
Non-uniform refinement (203,008 particles)
Local refinement (203,008 particles)
Sharpening
pri-RISC: 3.79 Å
Non-uniform refinement (205,047 particles)
Particle re-extraction (204,718 particles)
Reference motion correction (204,613 particles)
Ab-initio reconstructions (K=2)
Heterogeneous refinement (K=2)
Class #0 (161,301 particles)
Class #1 (43,312 particles)
3D classification (K=2)
Class #0 (85,751 particles)
Class #1 (75,550 particles)
Ab-initio reconstructions (K=2)
Heterogeneous refinement (K=2)
Ab-initio reconstructions (K=2)
Heterogeneous refinement (K=2)
Class #0 (64,361 particles)
Class #1 (21,390 particles)
Class #0 (11,206 particles)
Class #1 (64,344 particles)
Non-uniform refinement
Local refinement
Sharpening
Non-uniform refinement
Local refinement
Sharpening
vise-RISC: 3.87 Å
wedg-RISC: 3.77 Å

Figure S2. Reconstruction of pri-, vise-, and wedg-RISCs (related to Figs. 1 and 3)
Cryo-EM data processing workflow for pri-, vise-, and wedg-RISCs with CryoSPARC. A representative micrograph after patch motion correction is shown. Two data sets were collected from the same grid. One of the data sets was used to generate initial volumes. The extracted particles from both data sets were combined for the following processing.

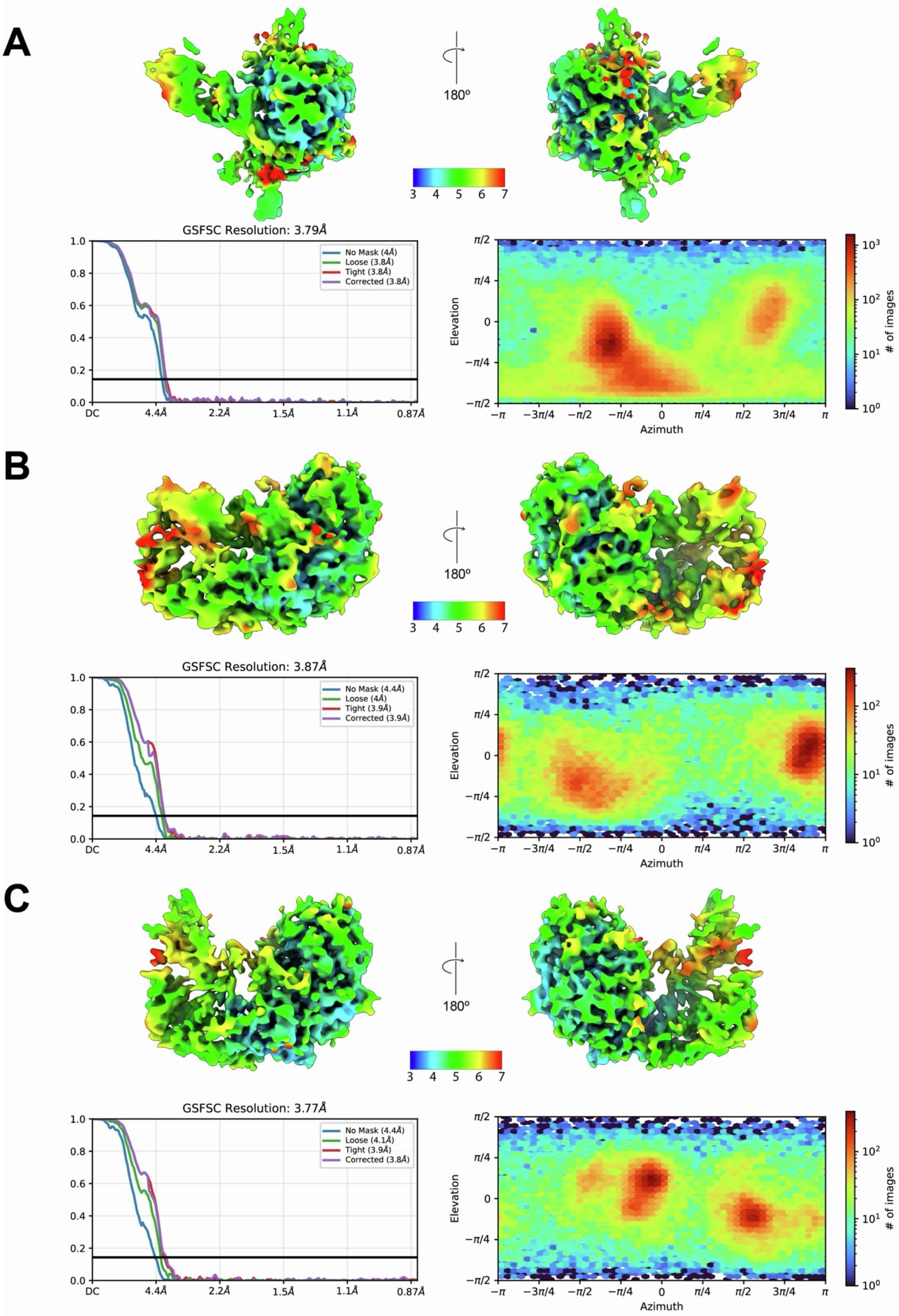

Figure S3. Qualities and resolutions of pri-, vise-, and wedg-RISCs (related to Figs. 1 and 3)
(A-C) Local resolutions of pri- (A), vise- (B), and wedg-RISC (C) maps with the corresponding FSC curves and viewing direction distribution heatmaps from CryoSPARC.

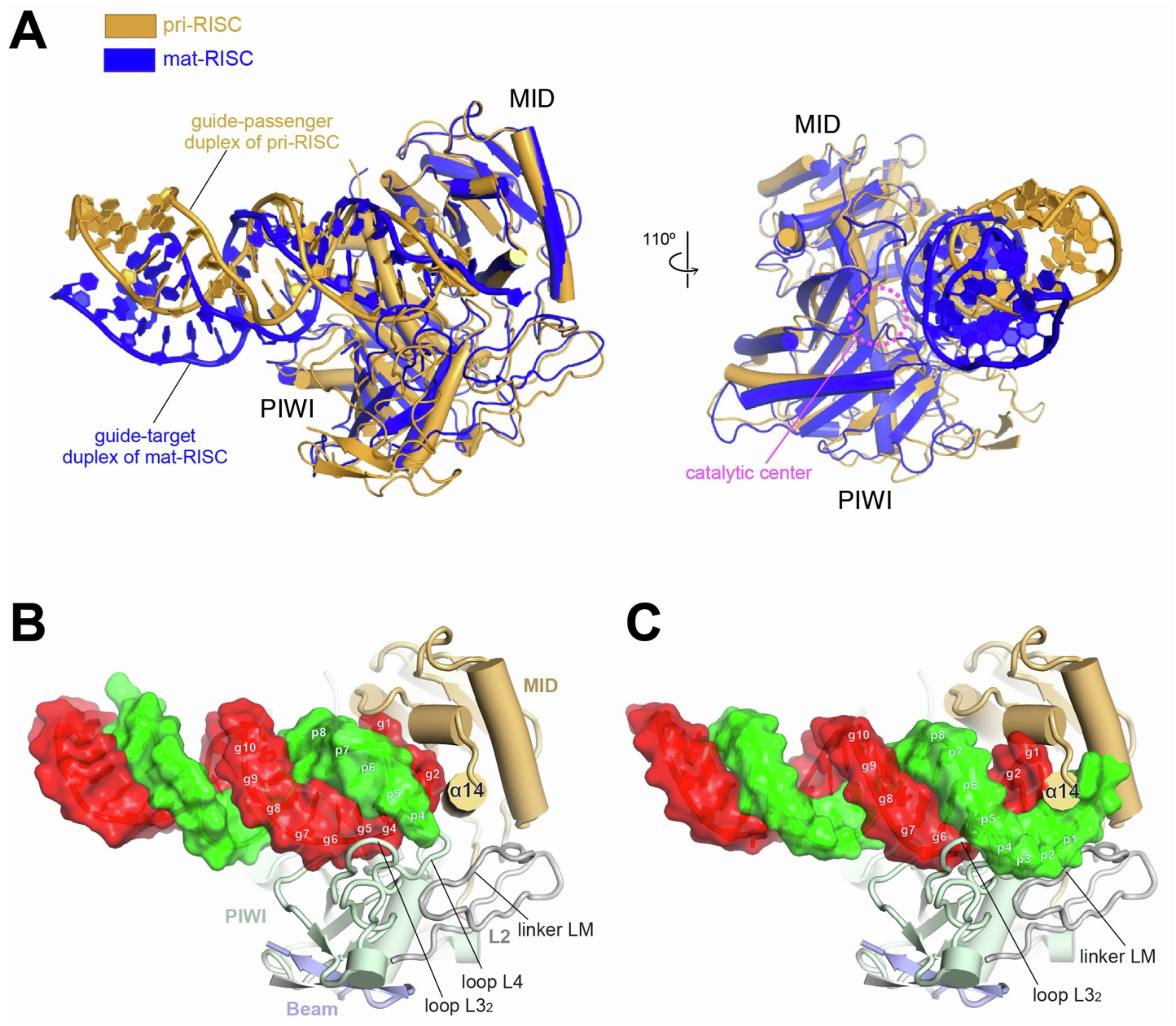

Figure S4. Structure of an open conformation of pri-RISC and the role of α14 (related to Figs. 1-2)
(A) Comparison of pri-RISC with mat-RISC in an open conformation. pri-RISC (gold) and a previously reported open conformation of AGO2 mat-RISC in complex with 21-nt target (blue, PDB ID: 9K6T) are superposed in their MID domains. pri-RISC differently arranges the PIWI domain and the duplex relative to the MID domain, unlike those of mat-RISC.

(B) Cryo-EM structure of pri-RISC. The AGO2 and siRNA duplex are shown as ribbon and surface models, respectively. The guide and passenger strands are colored red and green, respectively. The guide, g1-g10, and passenger, p1-p8, nucleotide positions are marked.

(C) A 23-nt ideal A-form RNA duplex (21-nt stem with 3′ 2-nt overhang) is superposed on the siRNA duplex of pri-RISC. The color codes are the same in (B).

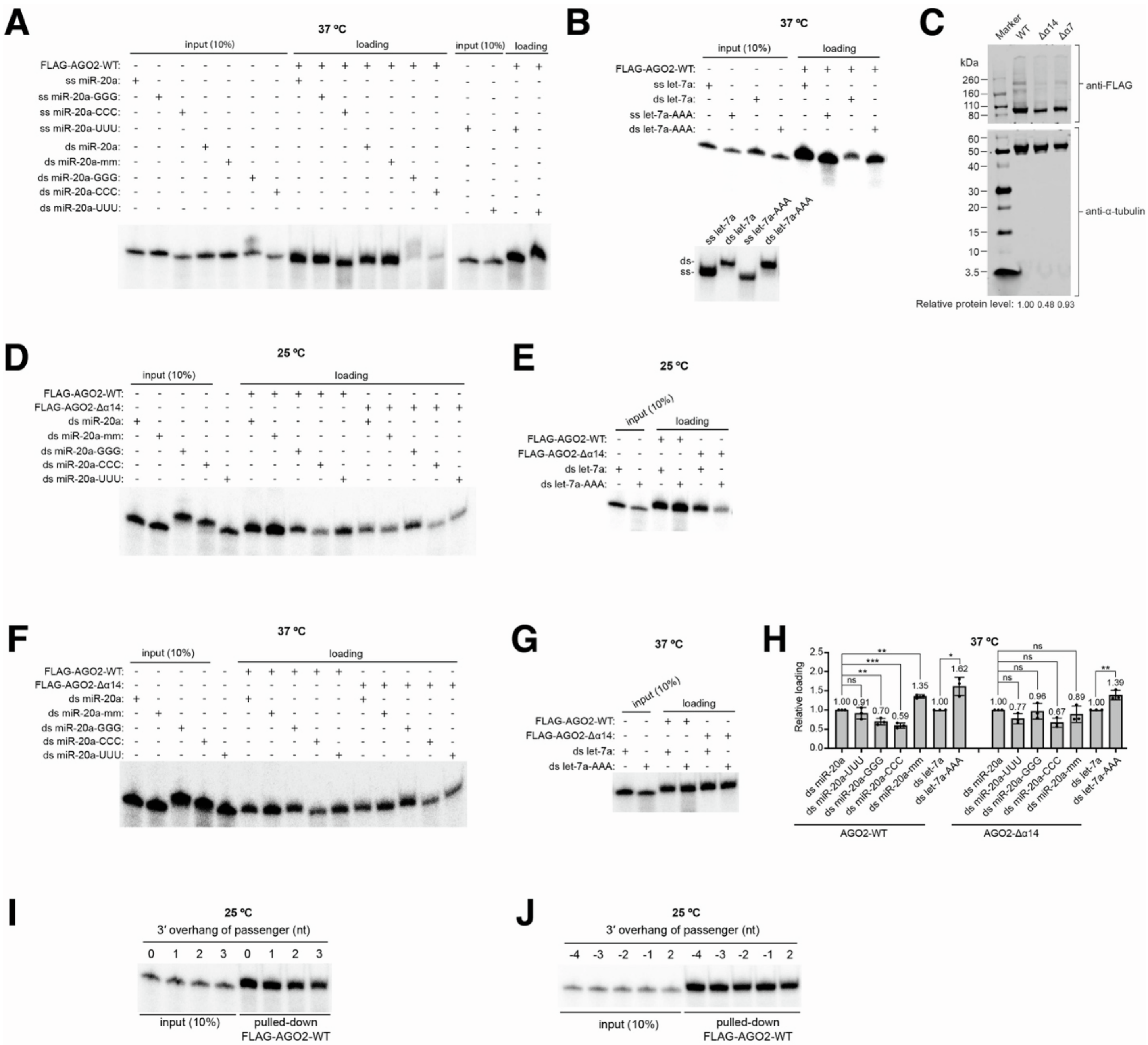

Figure S5. AGO2 alone preferentially loads the less stable end of siRNA duplex (related to Fig. 2)
(A) A representative 20% denaturing gel of in vitro loading assay using miR-20a guide. The designated ss and ds miR-20a and their variants were incubated with the purified recombinant FLAG-AGO2 at 37 °C. Half of the extracted RNA was resolved on this denaturing gel, while the other half was run on a 16% native gel, as shown in Fig. 2C.

(B) A representative 20% denaturing gel of in vitro loading assay using let-7a guide. The designated ss and ds let-7a and variants were incubated with the purified recombinant FLAG-AGO2 at 37 ºC. Half of the extracted RNAs were run on this denaturing gel (top), while the other half was run on a 16% native gel (bottom).

(C) Western blot assay of FLAG-AGO2-WT, -Δα14, and -Δα7 expressed in HEK293T cells. α-tubulin was used for normalization.

(D-G) Representative 20% denaturing gels of in vitro loading of the designated ss and ds miR-20a, let-7a, and variants into the immunopurified FLAG-AGO2-WT and -Δα14 from HEK293T cells at 25 ºC (D-E) or 37 ºC (F-G).

(H) Relative loading of designated miR-20a and let-7a into the immunopurified FLAG-AGO2-WT and -Δα14 from HEK293T cells at 37 ºC.

(I-J) Representative 20% denaturing gels of in vitro loading of the designated miR-20a duplex with different lengths of passenger 3' overhangs into purified recombinant FLAG-AGO2-WT at 25 ºC. Guide RNAs were 5' ^{32}P-labeled for all assays. All assays were triplicated.

Statistics were calculated using one-way ANOVA with Dunnett's post-hoc test. All assays were triplicated. Data are the mean ± SD, with *p*-values represented as follows: * $p < 0.05$, ** $p < 0.01$, *** $p < 0.001$, **** $p < 0.0001$, ns, not significant.

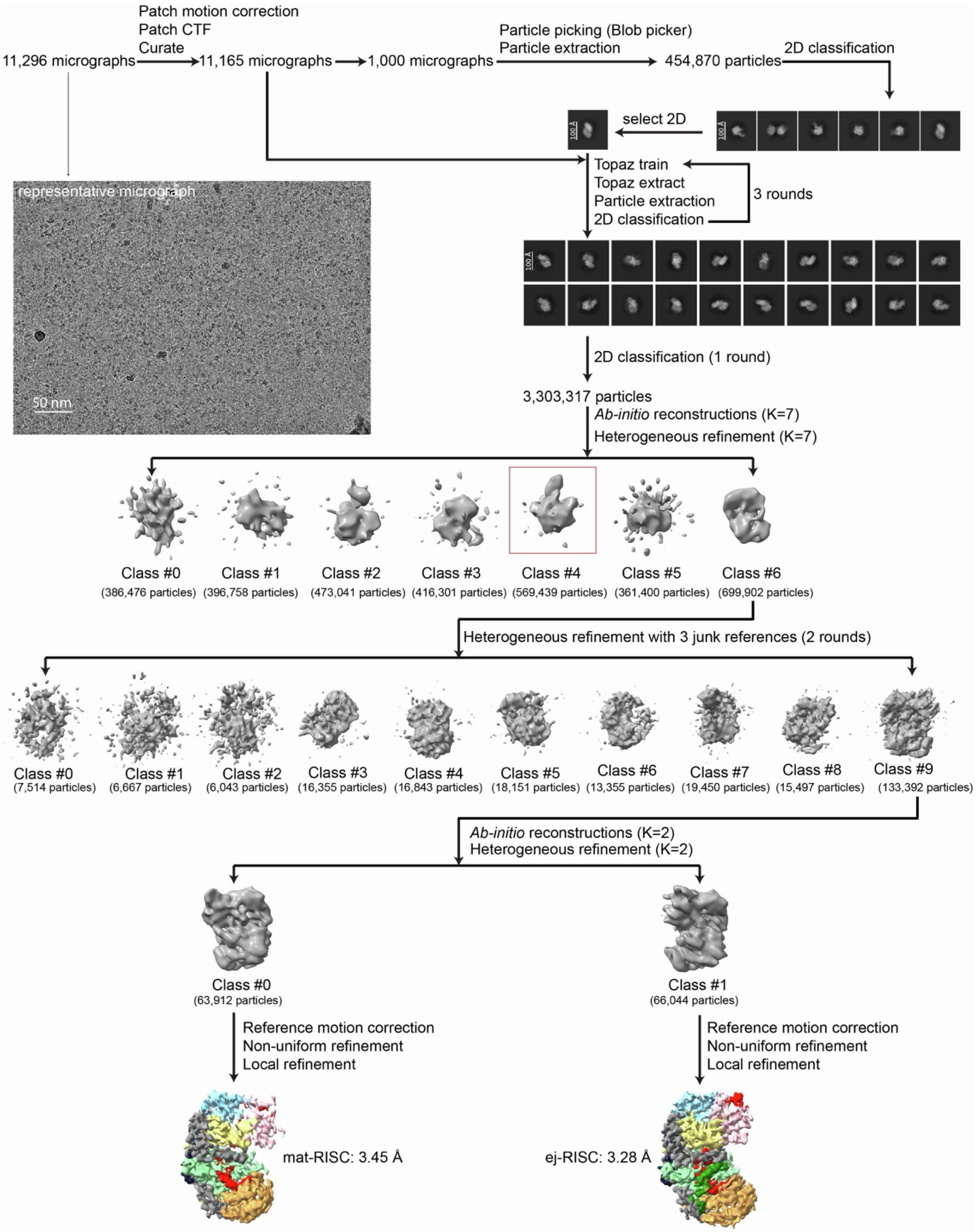

Figure S6. Reconstruction of ej and mat-RISCs with CryoSPARC (related to Figs. 4 and 5)
Cryo-EM data processing workflow for ej- and mat-RISCs with CryoSPARC. A representative micrograph after patch motion correction is shown. Class #4 highlighted in the red box indicates the class of pri-RISC as in Fig.S2.

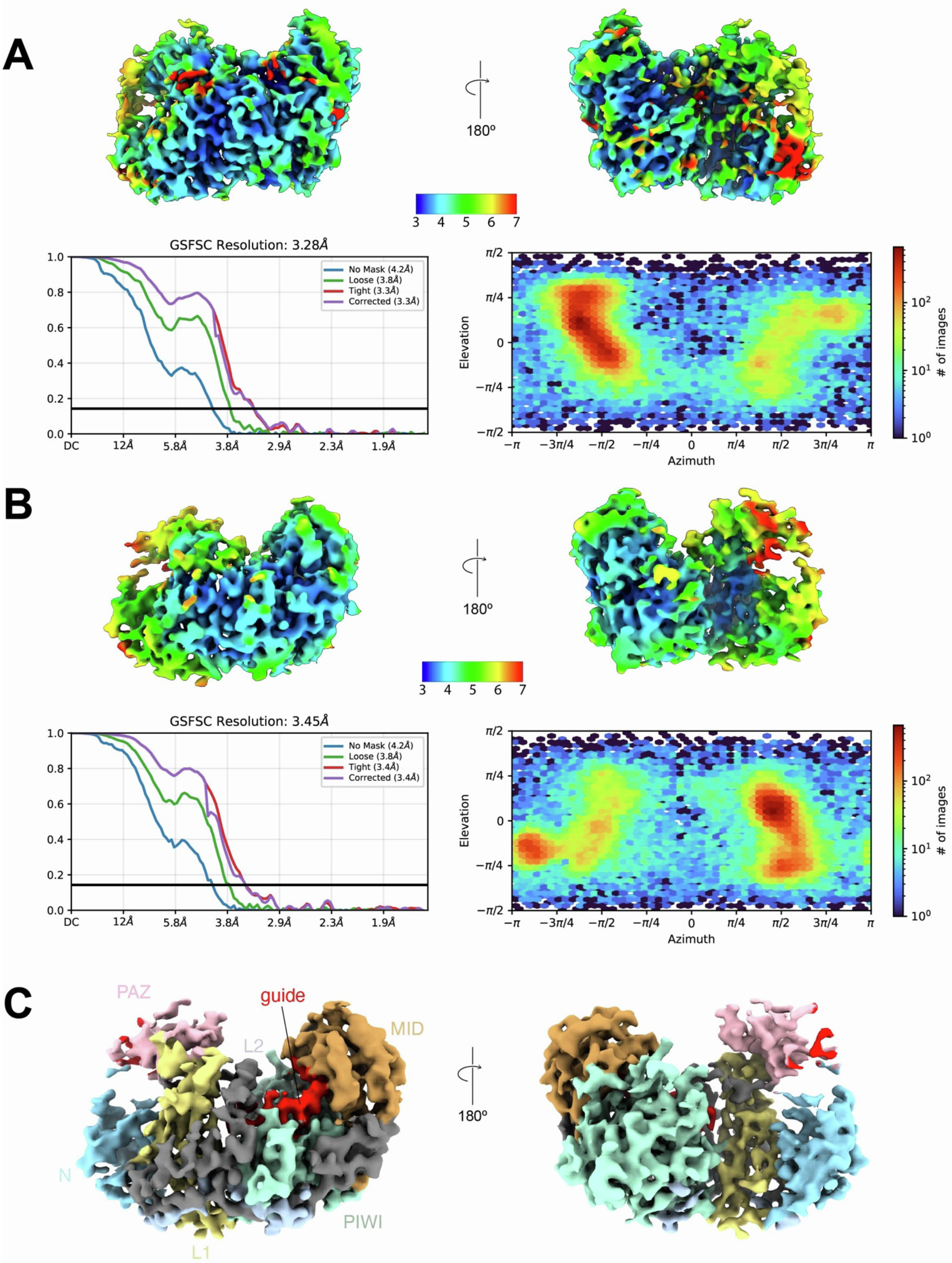

Figure S7. Qualities and resolutions of ej and mat-RISCs with CryoSPARC (related to Figs. 4 and 5)
(A-B) Local resolutions of ej- (A) and mat-RISC (B) maps with the corresponding FSC curves and viewing direction distribution heatmaps from CryoSPARC.

(C) Cryo-EM map of mat-RISC. The domain color codes are the same as shown in Fig. 4A. No passenger density was present.

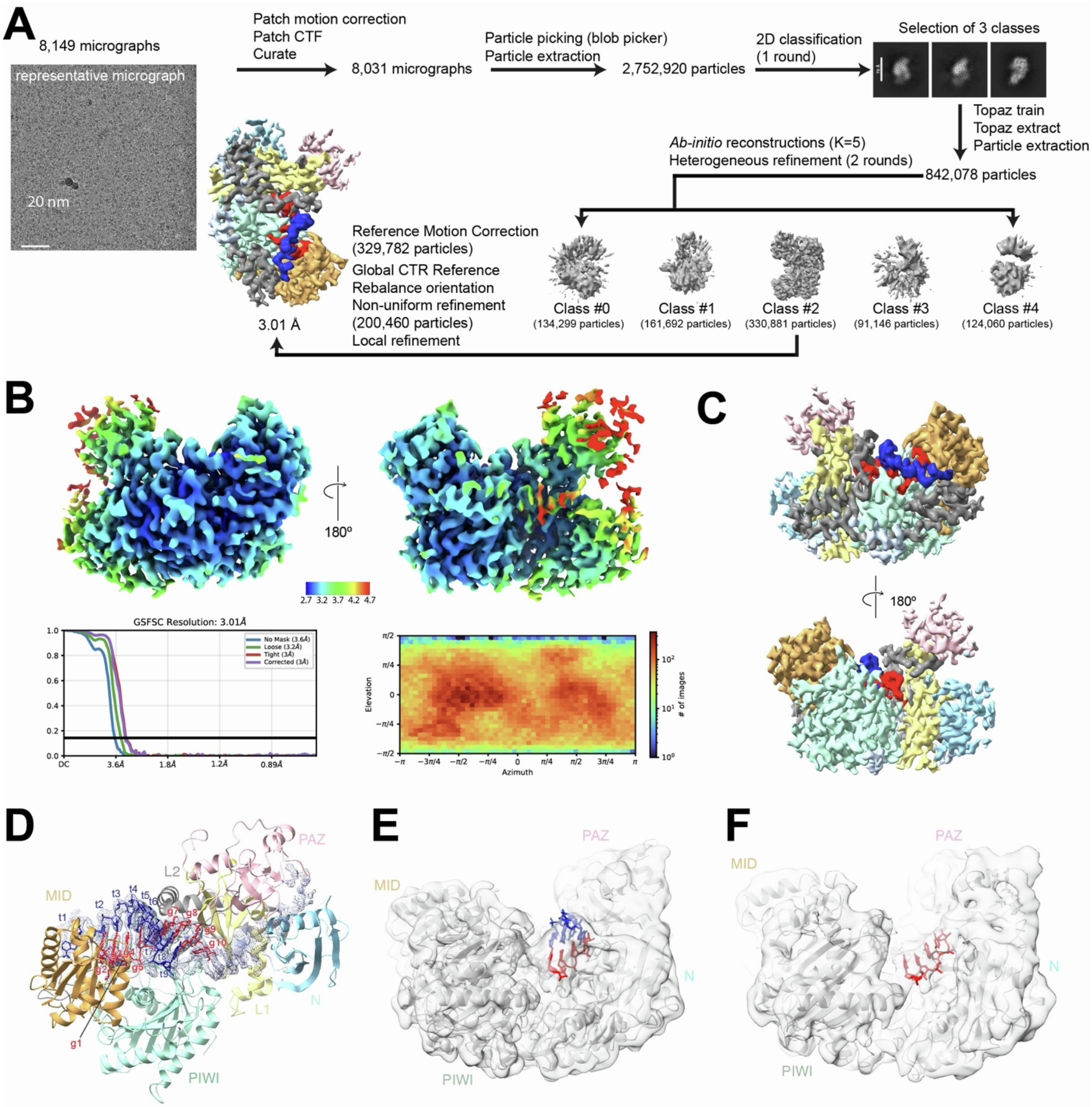

Figure S8. Reconstruction of targe-bound mat-RISC with CryoSPARC and the structural comparison with ej-RISC (related to Figs. 4)

(A) Cryo-EM data processing workflow for target-bound mat-RISC with CryoSPARC. A representative micrograph after patch motion correction is shown.

(B) Local resolution of target-bound mat-RISC maps with the corresponding FSC curve and viewing direction distribution heatmap from CryoSPARC.

(C) Cryo-EM map of target-bound mat-RISC. The domain color codes are the same as shown in Fig. 4a.

(D) Cryo-EM structure of target-bound mat-RISC. The color codes for the AGO2 domains are the same as shown in Fig. 4A, except that the target strand is colored in blue. The density for nucleic acids, shown as a mesh, indicates that the post-seed region is blurry.

(E-F) Low-pass Gaussian-filtered maps of target-bound mat-RISC (E) and ej-RISC (F). Only the target-bound mat-RISC shows a wide density capable of accommodating the 3' supplementary region of the guide (red) and the pairing target (blue).

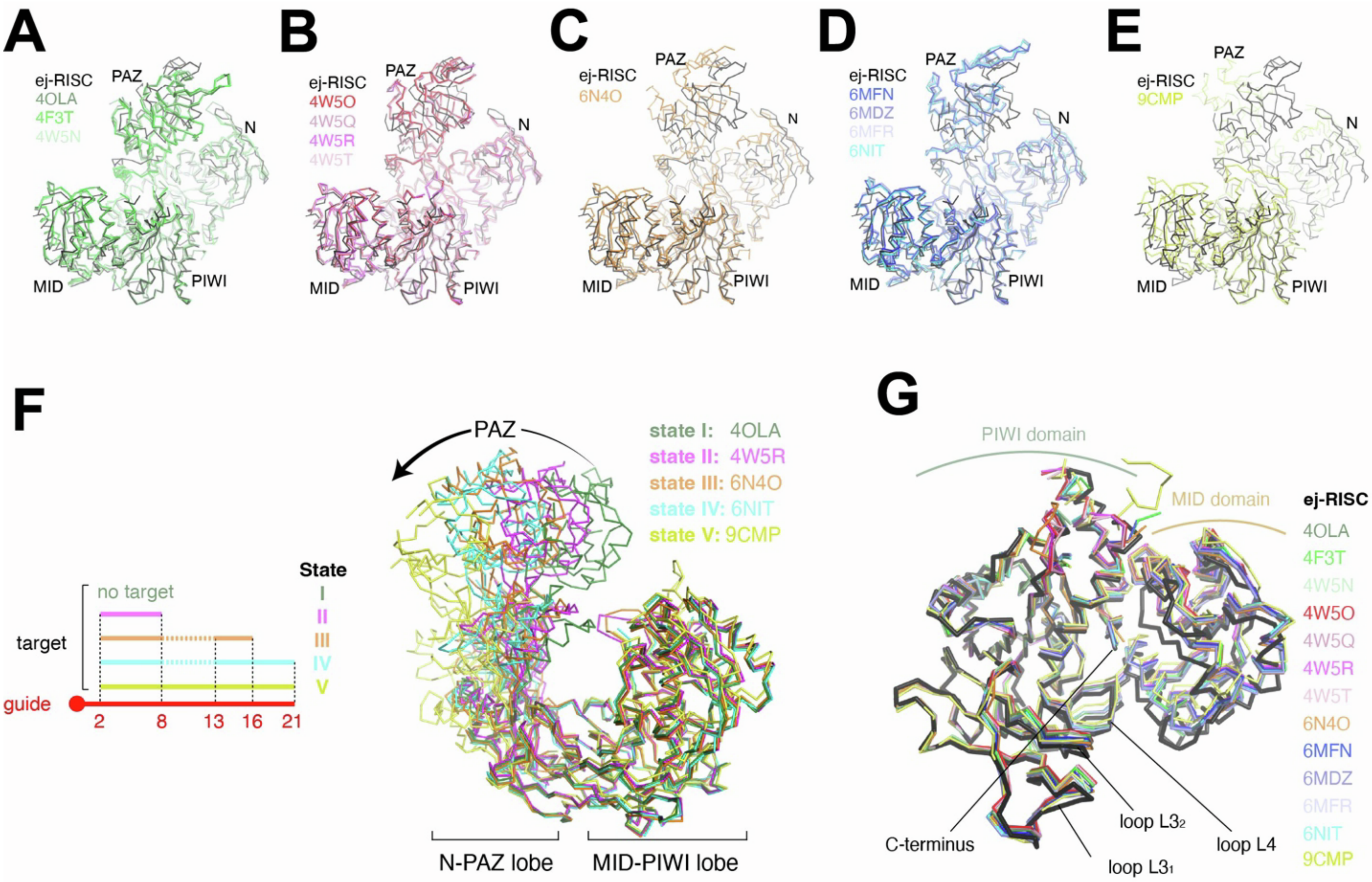

Figure S9. ej-RISC takes a different conformation from all previously reported structures of mat-RISC (related to Fig. 5)

(A-E) Superposition of ej-RISC and the previously determined AGO2 mat-RISCs in state I (A), II (B), III (C), IV (D), and V (E). Note that the states for pre- and mat-RISCs are written as lowercase (i, ii, iii, and iv; see Extended Data Fig. 1A) and capital (I, II, III, IV, and V), respectively. The model of ej-RISC colored black is overlaid on the PIWI catalytic cores (i.e., the PIWI-catalytic subdomain but excluding loops $L3_1$ and $L3_2$) of the AGO2 mat-RISCs. The PDB IDs and the corresponding models are shown in the same color. For clarification, the guide and target strands are not shown.

(F) AGO2 mat-RISC widening the two lobes from state I to V. (left) The guide-target pairing of each state is drawn. (right) One representative model is selected from each state, and they are superposed on the PIWI catalytic cores.

(G) AGO2 mat-RISCs shown in (A-E) are superposed on their PIWI catalytic core.

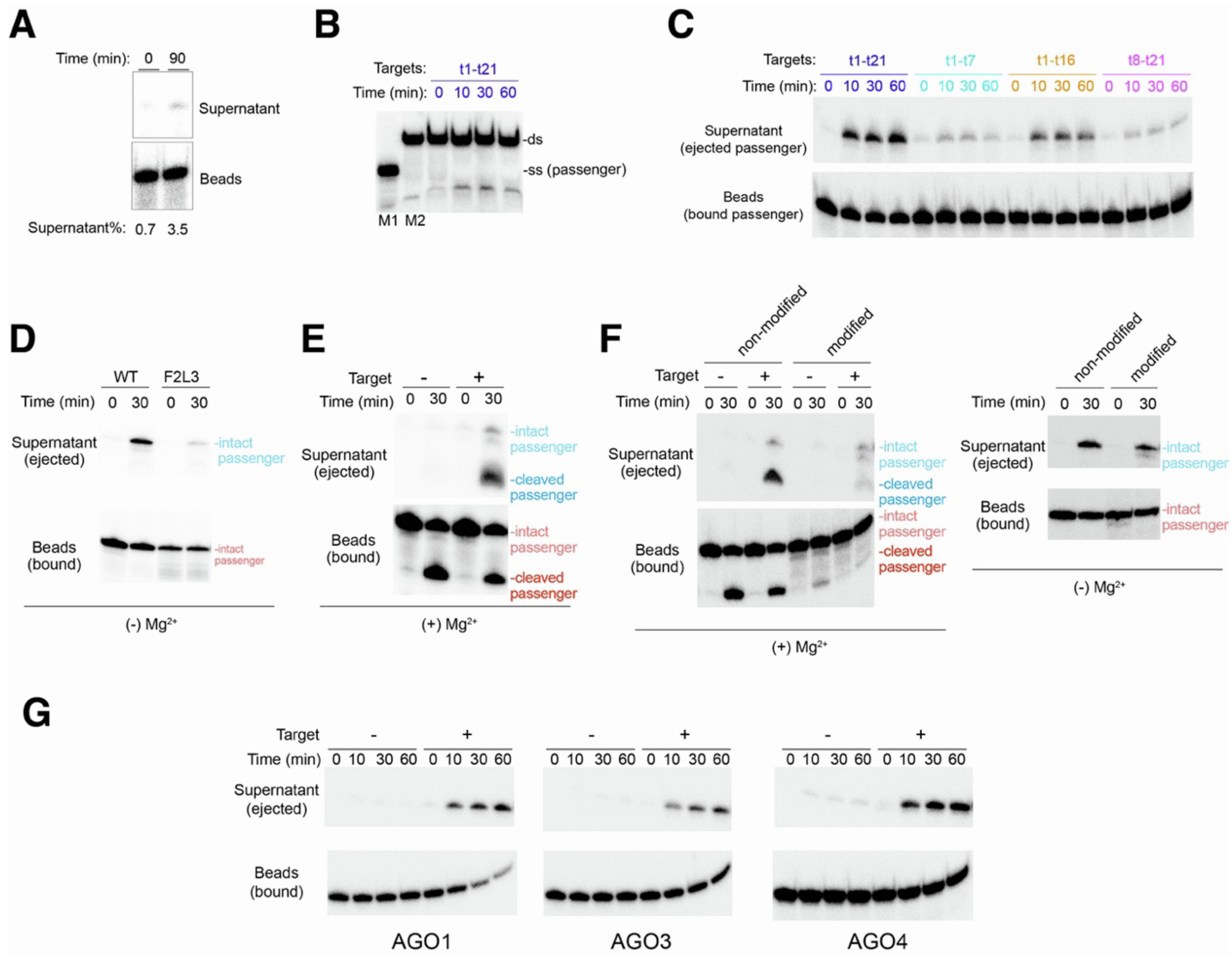

Figure S10. Target RNA facilitates passenger ejection (related to Fig. 6)
(A) A representative 20% denaturing gel of the guide RNA ejection assay. An siRNA-like duplex, composed of a 5' ^{32}P-labeled miR-20a guide strand and a cold passenger strand, was loaded onto purified FLAG-AGO2-WT. After extensive washes, a fully complementary target was added.

(B) A representative 16% native PAGE gel of passenger strand replacement by target RNA in the absence of AGO2. An siRNA-like duplex, composed of a cold miR-20a guide strand and a 3' ^{32}P-labeled passenger strand, was incubated with 40x target for the indicated time. M1 and M2 are ss and ds RNAs as markers.

(C) A representative 20% denaturing gel of the passenger ejection assay. The designated target RNAs were tested for TAPE.

(D) A representative 20% denaturing gel of the passenger ejection assay using AGO2 WT and F2L3 in the absence of Mg^{2+} and in the presence of a fully complementary target.

(E) A representative 20% denaturing gel of the passenger ejection assay using AGO2 WT in the presence of Mg^{2+} with and without the addition of a target.

(F) A representative 20% denaturing gel of the passenger ejection assay using AGO2 WT incubated with a non-modified or modified duplex. The modified duplex comprises a passenger strand with a phosphorothioate linkage between p10 and p11. The assay was performed either in the presence (left) or absence (right) of Mg^{2+}.

(G) A representative 20% denaturing gel of passenger ejection assays using human AGO1, AGO3, and AGO4 with and without a fully complementary target RNA.

All assays were performed at 37 ºC.

Table S1 Oligonucleotides used in the study (related to Fig. 1-6)

Oligonucleotide Name	Sequence	Source	Identifier
Biotinylated miR-20a passenger (5'-3')	Biotin-AAGAGACCUGCACUAUAAGCACUUUAAG	This paper	N/A
23-nt miR-20a guide (5'-3')	pUAAAGUGCUUAUAGUGCAGGUAG	This paper	N/A
23-nt miR-20a passenger (5'-3')	ACCUGCACUAUAAGCACUUUAAG	This paper	N/A
23-nt miR-20a-2-4mm passenger (5'-3')	ACCUGCACUAUAAGCACAAAAAG	This paper	N/A
23-nt miR-20a-UUU guide (5'-3')	pUUUUGUGCUUAUAGUGCAGGUAG	This paper	N/A
23-nt miR-20a-GGG guide (5'-3')	pUGGGGUGCUUAUAGUGCAGGUAG	This paper	N/A
23-nt miR-20a-GGG passenger (5'-3')	ACCUGCACUAUAAGCACCCCAAG	This paper	N/A
23-nt miR-20a-CCC guide (5'-3')	pUCCCGUGCUUAUAGUGCAGGUAG	This paper	N/A
23-nt miR-20a-CCC passenger (5'-3')	ACCUGCACUAUAAGCACGGGAAG	This paper	N/A
Modified 23-nt miR-20a passenger (5'-3')<br>(* indicates a phosphorothioate linkage)	ACCUGCACUAU*AAGCACUUUAAG	This paper	N/A
21-nt let-7a guide (5'-3')	pUGAGGUAGUAGGUUGUAUAGU	This paper	N/A
21-nt let-7a passenger (5'-3')	UAUACAACCUACUACCUCAGU	This paper	N/A
21-nt let-7a-AAA guide (5'-3')	pUAAAGUAGUAGGUUGUAUAGU	This paper	N/A
21-nt let-7a-AAA passenger (5'-3')	UAUACAACCUACUACUUUAGU	This paper	N/A
miR-20a passenger with 0-nt 3' overhang (5'-3')	ACCUGCACUAUAAGCACUUUA	This paper	N/A
miR-20a passenger with 1-nt 3' overhang (5'-3')	ACCUGCACUAUAAGCACUUUAA	This paper	N/A
miR-20a passenger with 3-nt 3' overhang (5'-3')	ACCUGCACUAUAAGCACUUUAAGU	This paper	N/A
miR-20a passenger with -1-nt 3' overhang (5'-3')	ACCUGCACUAUAAGCACUUU	This paper	N/A
miR-20a passenger with -2-nt 3' overhang (5'-3')	ACCUGCACUAUAAGCACUU	This paper	N/A
miR-20a passenger with -3-nt 3' overhang (5'-3')	ACCUGCACUAUAAGCACU	This paper	N/A
miR-20a passenger with -4-nt 3' overhang (5'-3')	ACCUGCACUAUAAGCAC	This paper	N/A
capture oligo (5'-3')<br>(m: 2'-O-methylation)	Biotin-<br>[mU][mC][mU][mU][mC][mG][mA][mU][mG][mG][mA]<br>[mC][mC][mA][mC][mU][mA][mA][mC][mA][mG][mC][<br>mA][mC][mU][mU][mU][mA][mA][mC][mC][mU][mU]	Zhang, et al.[1]	N/A
elution oligo (5'-3')	AAGGTTAAAGTGCTGTTAGTGGTCCATCGAAGA	Zhang, et al.[1]	N/A
miR-20a t1-t7 target (5'-3')	UAAUAAUAAUAUUCCACUUUAAG	This paper	N/A
miR-20a t8-t21 target (5'-3')	ACCUGCACUAUAAGAUGAAAAAG	This paper	N/A
miR-20a t1-t16 target (5'-3')	UAAUACACUAUAAGCACUUUAAG	This paper	N/A
miR-20a t1-t21 target (5'-3')	ACCUGCACUAUAAGCACUUUAAG	This paper	N/A
18-nt miR-20a target-2mm (5'-3')	CACUAACAGCACUUUAAA	This paper	N/A

1. Zhang, H., Sim, G., Kehling, A.C., Adhav, V.A., Savidge, A., Pastore, B., Tang, W., and Nakanishi, K. (2024). Target cleavage and gene silencing by Argonautes with cityRNAs. Cell Rep *43*, 114806. 10.1016/j.celrep.2024.114806.
